# Microtubule-sliding modules based on kinesins EG5 and PRC1-dependent KIF4A drive human spindle elongation

**DOI:** 10.1016/j.devcel.2021.04.005

**Published:** 2021-05-03

**Authors:** Kruno Vukušić, Ivana Ponjavić, Renata Buđa, Patrik Risteski, Iva M. Tolić

**Affiliations:** 1 Division of Molecular Biology, Ruđer Bošković Institute, Bijenička cesta 54, 10000 Zagreb, Croatia

**Keywords:** mitosis, anaphase, spindle elongation, motor proteins, microtubule sliding, functional redundancy, anaphase B, kinesins, EG5/kinesin-5, KIF4A/kinesin-4

## Abstract

Proper chromosome segregation into two future daughter cells requires the mitotic spindle to elongate in anaphase. However, although some candidate proteins are implicated in this process, the molecular mechanism that drives spindle elongation in human cells is unknown. Using combined depletion and inactivation assays together with CRISPR technology to explore redundancy between multiple targets, we discovered that the force-generating mechanism of spindle elongation consists of EG5/kinesin-5 together with the PRC1-dependent motor KIF4A/kinesin-4, with contribution from kinesin-6 and kinesin-8. Disruption of EG5 and KIF4A leads to total failure of chromosome segregation due to blocked spindle elongation, despite poleward chromosome motion. Tubulin photoactivation, stimulated emission depletion (STED), and expansion microscopy show that perturbation of both proteins impairs midzone microtubule sliding without affecting microtubule stability. Thus, two mechanistically distinct sliding modules, one based on a self-sustained and the other on a crosslinker-assisted motor, power the mechanism that drives spindle elongation in human cells.

## Highlights

• Spindle elongation depends on the joint activity of EG5/kinesin-5 and PRC1
• PRC1-dependent KIF4A/kinesin-4 is crucial for elongation upon EG5 inhibition
• EG5 and KIF4A together slide antiparallel microtubules during early anaphase
• Spindle elongation block induces large chromosome segregation defects

## Graphical abstract

**Figure:**
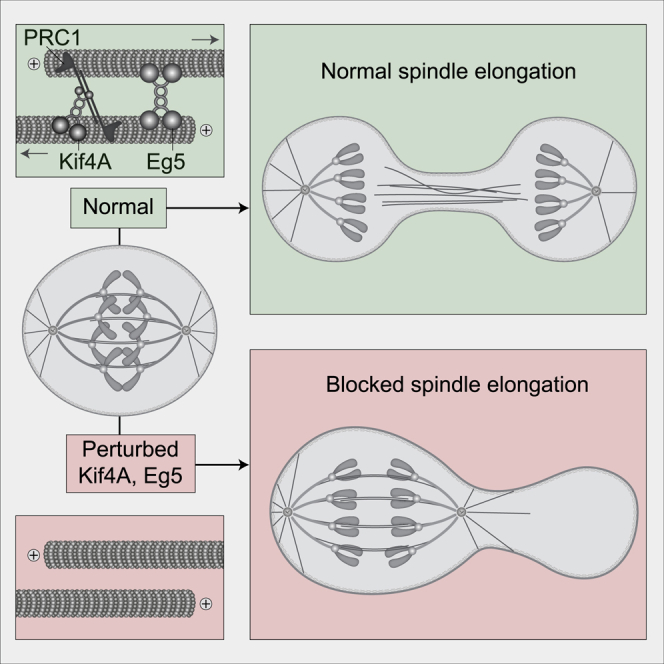

## Teaser

Vukušić et al. demonstrate that spindle elongation in human cells is powered by joint activity of kinesin-5 and kinesin-4 through their role in antiparallel microtubule sliding. Without elongation chromosome segregation frequently fails, demonstrating its requirement for successful cell division.

## Introduction

Cell division is a fundamental process required for stable transmission of genetic information from a parent cell to two daughter cells. Sister chromatids segregate into future daughter cells during anaphase by kinetochore fiber shortening (anaphase A) and spindle elongation (anaphase B) (McIntosh et al., 2012; Maiato and Lince-Faria, 2010). Although different organisms vary in the relative contribution of strategies used for effective chromosome segregation (Asbury, 2017; Scholey et al., 2016), spindle elongation is omnipresent during anaphase in eukaryotes, from fungi to humans (Roostalu et al., 2010). Interestingly, even plasmid separation in prokaryotes shares many common features with anaphase B (Gayathri et al., 2012). The importance of spindle elongation in human cells is evident from the fact that in addition to being the main driver of chromosome movement (Su et al., 2016), it promotes correct segregation of lagging chromosomes (Cimini et al., 2004), and its defects are correlated with cancer (Ford, 2013).

**Figure 1:**
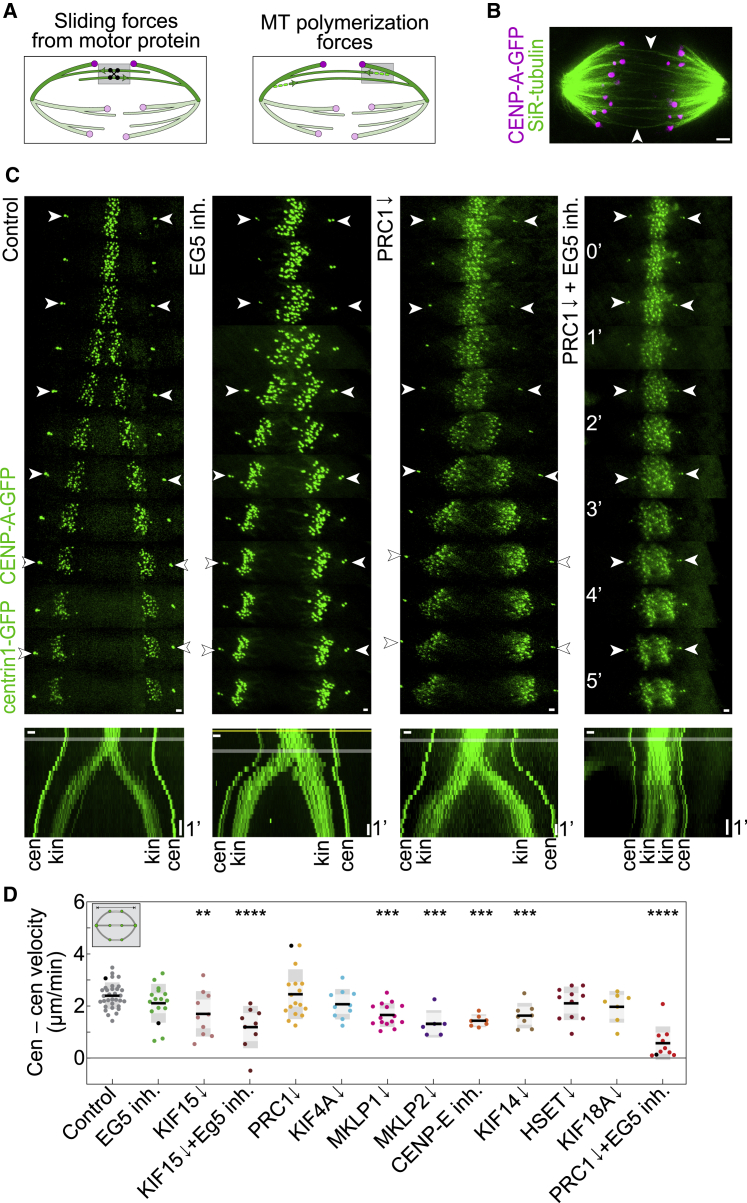
Depletion of PRC1 and inactivation of EG5 block spindle elongation during anaphase (A) Two models of spindle elongation. (B) STED image (single z plane) of an anaphase spindle in a live U2OS cell expressing CENP-A-GFP (magenta). Microtubules are labeled with 100-nM SiR-tubulin (green). Arrowheads point to the spindle midzone where antiparallel microtubules are located. (C) Live-cell images (top) and corresponding kymographs (bottom) of control, STLC-treated (EG5 inh.), PRC1-siRNA-depleted (indicated by an arrow pointing down) and PRC1-siRNA-depleted 40-μM STLC-treated RPE-1 cells stably expressing CENP-A-GFP and centrin1-GFP. kin, kinetochore; cen, centrosome. White arrowheads indicate centrioles. Horizontal gray lines in the kymographs indicate the onset of anaphase. (D) Quantification of spindle elongation velocity (see scheme) (n = 34, 16, 10, 9, 17, 10, 15, 6, 7, 8, 11, 7, and 10 cells, from left to right), measured in the period from the 1–3 min after anaphase onset for each treatment. More than three independent experiments for every condition regarding siRNAs or non-targeting treatments, while number of independent experiments regarding STLC treatment is equal to the number of cells. Boxes represent standard deviation (dark gray), 95% confidence interval of the mean (light gray), and mean value (black). Black data dots in every treatment correspond to the measurements from the exemplar cells shown on the time-lapse images and kymographs (C). Statistics: t test (^∗^p < 0.05; ^∗∗^p < 0.01; ^∗∗∗^p < 0.001; ^∗∗∗∗^p < 0.0001). Time is shown as minutes. Images are the maximum projection of the acquired z stack. Time 0 represents anaphase onset. Horizontal scale bars, 1 μm. Vertical scale bars, 1 min. See also Figure S1.

Two landmark models for anaphase spindle elongation have been proposed: in the “midzone pushing model” overlap microtubules generate pushing forces from the spindle midzone, whereas in the “cortical puling model” astral microtubules generate forces by interacting with the cell cortex (Scholey et al., 2016). Although cortical pulling forces may contribute during late anaphase (Pamula et al., 2019; Kiyomitsu and Cheeseman, 2013; Kotak et al., 2013), recent work suggests that the spindle in human cells elongates predominantly by pushing forces from the spindle midzone, generated by sliding apart of antiparallel microtubules (Figures 1A and 1B) (Vukušić et al., 2019, 2017; Yu et al., 2019). Sliding in the midzone may be driven by motor proteins in the antiparallel overlaps, which is known as the “sliding filament mechanism” (Figure 1A, left) (McIntosh et al., 1969), and by microtubule growth against barriers (Figure 1A, right) (Laband et al., 2017; Dogterom et al., 2005). While anaphase A is thought to rely on the microtubule depolymerization-driven mechanisms, where motors are regulators of microtubule dynamics or couplers of kinetochore movement to microtubules (Asbury, 2017), anaphase B is thought to be highly motor-dependent process (Scholey et al., 2016; Cande, 1982). Identification of plus-end-directed mitotic motors (Kashina et al., 1996; Nislow et al., 1992) and *in vitro* experiments that established microtubule-sliding capacity of numerous motors (Wijeratne and Subramanian, 2018; Su et al., 2013; Kapitein et al., 2005; Nislow et al., 1992) fueled the search for proteins that drive spindle elongation in human cells. However, despite extensive work that mainly relied on depletions or inhibitions of individual mitotic proteins (Vukušić et al., 2017; Su et al., 2016; van Heesbeen et al., 2014; Kiyomitsu and Cheeseman, 2013; Hu et al., 2011; Zhu et al., 2005; Nislow et al., 1992), the molecular mechanism of spindle elongation in human cells remained a longstanding open question.

Here, we developed combined depletion and inactivation assay together with CRISPR technology to systematically explore the redundancy between motor proteins during anaphase B. We found that the force-generating mechanism of spindle elongation consists of two plus-end-directed motors that work together, the PRC1-dependent motor KIF4A/kinesin-4 and EG5/KIF11/kinesin-5. Unexpectedly, disruption of activity of both motors leads to failed chromosome segregation due to blocked spindle elongation, despite continuous poleward chromosome motion. By using tubulin photoactivation and super-resolution microscopy, we show that perturbation of both proteins directly impairs midzone microtubule sliding without disrupting microtubule stability. Thus, two independent microtubule-sliding modules, one based on a self-sustained EG5 tetrameric motor and the other on a PRC1-assisted dimeric KIF4A motor, drive spindle elongation in human cells by a midzone pushing mechanism. Finally, as combined depletion of kinesins-6 was also modulated further by EG5 inhibition, similar to KIF4A depletion, this demonstrates the functional cooperation between crucial components of the cytokinetic machinery, PRC1-KIF4A-MKLP1-MKLP2, and the main pre-anaphase force generator EG5 for spindle elongation during anaphase.

## Results

### Anaphase spindle elongation is independent of individual activities of EG5/kinesin-5 and PRC1-dependent motors

To elucidate the molecular mechanisms of anaphase spindle elongation in human cells, we studied centrosome and kinetochore separation dynamics under loss-of-function treatments during early anaphase (first 5 min) because this is the period of major chromosome segregation in human cells (Su et al., 2016). Whereas spindle elongation can be altered by non-specific perturbations including changing temperature (Afonso et al., 2014), global microtubule dynamics (Hu et al., 2011), or interfering with top signaling effectors (Afonso et al., 2019; Brennan et al., 2007), we set out to identify the force-producing proteins whose perturbation stops spindle elongation during anaphase. We started with the perturbation of plus-end-directed motor EG5/kinesin-5, which is considered as a major candidate for anaphase spindle elongation, based on *in vitro* work demonstrating its sliding capacity, the notion that it can inhibit elongation when locked in rigor-bound state to midzone microtubules and the role of the kinesins-5 during yeast anaphase (Afonso et al., 2014; Collins et al., 2014; Kapitein et al., 2005; Straight et al., 1998). However, its role in anaphase spindle elongation in higher eukaryotes has not been demonstrated. We found that EG5 inhibition during live imaging of late metaphase cells by a small molecule drug S-trityl-L-cysteine (STLC) (Skoufias et al., 2006) in RPE-1 human cells did not stop spindle elongation during anaphase, and the velocity of spindle elongation was not significantly different when compared with untreated cells (Figures 1C, 1D, and S1A–S1C; Video S1). Also, depletion of kinesin-12/KIF15 (Tanenbaum et al., 2009) with a small interfering RNAs (siRNAs), alone or in combination with EG5 inhibition, did not stop spindle elongation, although the velocity of elongation was reduced compared with controls after both single depletion of KIF15 and when combined with inhibition of EG5 (Figures 1D and S1A–S1D). Moreover, inhibition of EG5, with or without KIF15 depletion, induced the delay in the start of anaphase B with respect to anaphase A, when compared with untreated cells (Figure S1E). Spindle length at the anaphase onset correlated positively with the spindle elongation velocity across conditions (Figure S1F), as in yeast spindles (Krüger et al., 2019). Interestingly, metaphase spindles quickly shortened or completely collapsed after STLC treatment (Figures S1G–S1J) (Gayek and Ohi, 2014), indicating that EG5-generated outward force is crucial during metaphase (van Heesbeen et al., 2014), but dispensable during anaphase when inward-forces acting on poles are turned down. This suggests involvement of force generators other than EG5 during anaphase in human cells.

Video S1. Anaphase spindle elongation is abrogated after depletion of PRC1 by siRNA and inhibition of EG5 by addition of STLC, related to Figure 1 Swept-field confocal time-lapse imaging of RPE-1 cells stably expressing CENP-A-GFP and centrin1-GFP under the indicated experimental conditions. Cells were recorded every 15 s. Time, s.

To test the role of other midzone proteins, we depleted the main crosslinker of antiparallel microtubules and scaffold for recruitment of multiple mitotic motors, protein regulator of cytokinesis 1 (PRC1) (Lee et al., 2012), by using siRNA, which resulted in a 98% decrease of the immunofluorescence intensity of PRC1 in the spindle midzone (Figures S2A and S2B). PRC1 depletion did not affect spindle elongation in early anaphase (Figures 1C, 1D, and S1A–S1C; Video S1), though it resulted in longer spindles in late anaphase when compared with control cells (Figure S2C) (Pamula et al., 2019). Accordingly, individual depletions by siRNA approach or inhibitions of all PRC1-interacting motor proteins, KIF4A/kinesin-4 (Kurasawa et al., 2004), KIF23/MKLP1/kinesin-6 (Figure S2D), KIF20A/MKLP2/kinesin-6 (Gruneberg et al., 2006), centromere-associated protein E (CENP-E)/kinesin-7 (Kurasawa et al., 2004), and KIF14/kinesin-3 (Gruneberg et al., 2006), did not stop spindle elongation, although all except for KIF4A significantly slowed down spindle elongation when compared with control cells (Figures 1D and S1A–S1D). To assess the level of protein depletion in experiments with siRNA targeting KIF4A, MKLP1, MKLP2, and KIF14, we quantified their immunofluorescence intensity in the midzone region and observed more than 80% decrease for all studied depletions when compared with non-treated cells (Figures S2A and S2B). This analysis of protein depletion specifically on anaphase spindles was corroborated by western blot analysis of the overall depletion of PRC1 and its major interacting partners (Figure S2E).

Similarly, efficient depletion of KIF18A/kinesin-8 by 24 h siRNA treatment (Figures S2A and S2B), a microtubule dynamics regulator, which localizes to the antiparallel bridging microtubules that link sister k-fibers during metaphase (Jagrić et al., 2021; Kajtez et al., 2016), early (Figure S2A) and late anaphase (Stumpff et al., 2008), and slides microtubules in yeast (Su et al., 2013), did not impact spindle elongation during early anaphase (Figures 1D and S1A–S1C). Also, efficient depletion of the minus-end-directed HSET/KIFC1/kinesin-14 (Figures S2A and S2B), previously implicated in the maintenance of stable midzone structure during cytokinesis (Cai et al., 2010), did not affect spindle elongation (Figures 1D and S1A–S1C). Thus, we argue that neither the EG5-KIF15 partnership, which is crucial for the maintenance of spindle length during metaphase, nor any single tested midzone-localized protein accounts for spindle elongation in human cells.

### Spindle elongation is driven by redundant EG5 and PRC1 protein modules

As individual depletion of midzone motors did not block spindle elongation, we hypothesized that two or more motors may act in a functionally redundant manner. First, we investigated whether PRC1 and EG5 are independent from each other during anaphase. We found that EG5 localizes to midzone microtubules after depletion of PRC1 (Figure S2F), suggesting that EG5 function in the midzone does not depend on PRC1. Similarly, PRC1 was present in the midzone after acute inhibition of EG5 (Figure S2G), showing that PRC1 localization does not require EG5 activity. To explore possible redundancy between PRC1-dependent motors and EG5, we inhibited EG5 at metaphase-to-anaphase transition in PRC1 depleted cells. Surprisingly, this prevented spindle elongation (spindle elongation velocity 0.55 ± 0.19 versus 2.36 ± 0.08 μm/min in control cells) resulting in unsuccessful chromosome segregation (Figures 1C, 1D, and S1A–S1C; Video S1), regardless if STLC was added in metaphase (Figure 1C) or early anaphase (Figure S2H). The effect was mostly restricted to spindle elongation (anaphase B), because the movement of chromosomes toward spindle poles (anaphase A) was not stopped, although it was slower compared with control cells (Figures 1C and S2I), suggesting that EG5 and PRC1 act primarily on anaphase B mechanisms.

**Figure 2:**
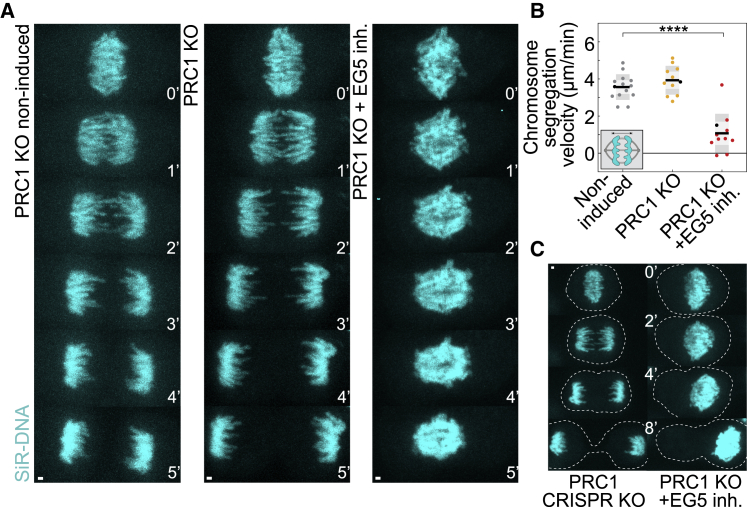
Chromosome segregation is compromised after KO of PRC1 and inhibition of EG5 (A) Live images of RPE-1 cells in PRC1 non-induced KO, induced PRC1 CRISPR KO and induced PRC1 KO treated with 40-μM STLC imaged 4 days after doxycycline induction. 100-nM SiR-DNA (cyan) was used for chromosome staining. (B) Quantification of chromosome segregation velocity (see scheme) in CRISPR experiments (n = 14, 11, and 11, from left to right). Two independent experiments for every condition except PRC1 KO + Eg5 inh. which was done in three independent experiments, while number of independent experiments regarding STLC treatment is equal to the number of cells. Black data dots in every treatment correspond to the measurements from the exemplar cells shown on the time-lapse images and kymographs (A). Statistics: t test (^∗^p < 0.05; ^∗∗^p < 0.01; ^∗∗∗^p < 0.001; ^∗∗∗∗^p < 0.0001). (C) Live-cell images of induced RPE-1 PRC1 CRISPR KO and induced PRC1 KO treated with 40-μM STLC. Dashed lines designate the cell borders. 100-nM SiR-DNA was used for chromosome staining. Time is shown as minutes. Images are maximum projections of the acquired z stack. Time 0 represents anaphase onset. Scale bars, 1 μm. See also Figure S2.

To check that the observed effect was not specific to the siRNA depletion approach and to completely remove PRC1 from a cell, we used inducible CRISPR PRC1 knockout (KO) cell line (McKinley and Cheeseman, 2017). To assess the efficiency of KO, we quantified PRC1 signal on anaphase spindles (Figure S2J) and found that 10% of cells showed normal PRC1 signal, implying that the efficiency of PRC1 KO was approximately 90% (n = 68 cells, see STAR Methods). High efficiency of PRC1 KO was confirmed by western blot analysis 5 days after KO induction, in comparison with non-induced control cells (Figure S2K). We observed normal chromosome segregation in non-induced control cells (Figures 2A, 2B, S2L, and S2M; Video S2) and similar velocities of chromosome segregation during early anaphase in induced PRC1 KO cells (Figures 2A, 2B, and S2L; Video S2). Furthermore, PRC1 KO cells were characterized by a hyper-segregation phenotype when compared with non-induced controls (Figures 2A, 2B, and S2L; Video S2), similar to previous observations after PRC1 shRNA in human HeLa cells (Pamula et al., 2019) and our observations after PRC1 siRNA in RPE-1 cells (Figure S2C). In contrast, chromosome segregation was completely impaired after EG5 inhibition by STLC addition in PRC1 KO cells, with ∼80% reduction in chromosome segregation velocities compared with non-induced control cells (Figures 2A, 2B, S2L, and S2M; Videos S2 and S3). This impairment was similar to the block of spindle elongation that resulted in perturbed kinetochore separation observed after PRC1 depletion by siRNA combined with EG5 inhibition (Figures 1C and 1D).

Video S2. Anaphase chromosome segregation is impaired after knockout (KO) of PRC1 by CRISPR-Cas9 and inhibition (inh.) of EG5 by addition of STLC, related to Figure 2 Swept-field confocal time-lapse imaging of RPE-1 cells labeled with 100-nM silicone rhodamine (SiR)-DNA under the indicated experimental conditions. Cells were recorded every 15 s. Time, s.

Video S3. Abrogation of chromosome segregation seen after EG5 inhibition (inh.) in PRC1 knockout (KO) is not specific to chromosome labeling with SiR-DNA, related to Figure 2 Brightfield time-lapse imaging of unlabeled RPE-1 cells under the indicated experimental conditions. Cells were recorded every 15 s. Time, s

Interestingly, EG5 inhibition in PRC1 KO cells often led to total failure of chromosome segregation characterized by two distinct segregation failure phenotypes (Figures 2C and S2N). In the first scenario, whole chromosome mass in some instances was transferred to one daughter cell at the time when the cleavage furrow started forming. In the second scenario, after chromosome segregation occurred to some degree, the portion of chromatin was left in the forming cleavage furrow (Figures 2C and S2N). Thus, the redundant activity of EG5 and PRC1 protein modules is critical for spindle elongation and proper chromosome segregation in human cells.

### The plus-end-directed motor proteins KIF4A and EG5 are crucial for spindle elongation

**Figure 3:**
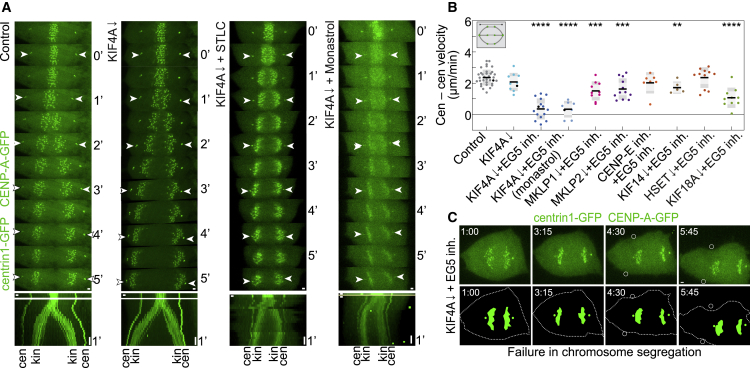
Depletion of KIF4A and inactivation of EG5 induce a block in chromosome segregation by blocking spindle elongation (A) Live-cell images of control, KIF4A siRNA-depleted, KIF4A-siRNA-depleted 40-μM STLC-treated, and KIF4A-siRNA-depleted 100-μM monastrol-treated RPE-1 cells stably expressing CENP-A-GFP and centrin1-GFP. kin, kinetochore; cen, centrosome. White arrowheads indicate centrioles. Horizontal gray bars in the kymographs indicate the onset of anaphase. Time zero represents anaphase onset. Time is shown as minutes. (B) Quantification of velocity of spindle elongation (n = 34, 10, 15, 8, 10, 12, 7, 6, 11, and 10 cells, from left to right). Three independent experiments for every condition regarding siRNAs or non-targeting treatments, while number of independent experiments regarding STLC treatment is equal to the number of cells. Black data dots in every treatment correspond to the measurements from the exemplar cells shown on the time-lapse images and kymographs (A). Statistics: t test (^∗^p < 0.05; ^∗∗^p < 0.01; ^∗∗∗^p < 0.001; ^∗∗∗∗^p < 0.0001). (C) Live images of KIF4A siRNA-depleted STLC-treated RPE-1 cell show complete failure in chromosome segregation after STLC addition during early anaphase. Time zero represents the start of STLC treatment. White circles represent the locations of the initiation of the cytokinetic cleavage furrowing. Time is shown as minutes:seconds. Images are the maximum projection of the acquired z stack. Horizontal scale bars, 1 μm. Vertical scale bars, 1 min. See also Figure S3.

As PRC1 is a passive microtubule bundler (Mollinari et al., 2002), we hypothesized that its necessity for spindle elongation is due to the recruitment of active motor proteins. To test this idea, we depleted PRC1-interacting motors, one by one, in combination with EG5 inhibition. Surprisingly, depletion of KIF4A by siRNA approach together with EG5 inhibition completely mirrored the blocked chromosome segregation phenotype deprived of spindle elongation (spindle elongation velocity 0.35 ± 0.17 versus 2.36 ± 0.08 μm/min in control cells) found after PRC1 depletion and EG5 inhibition (Figures 3A, 3B, S1A, and S3A–S3D; Video S4). This double perturbation resulted in ∼60% reduction of kinetochore separation velocity when compared with control cells (Figure S3D), even though poleward movement was still present albeit at a reduced rate compared with controls (Figures 3A and S3E). Moreover, double perturbation often resulted in catastrophic failure of chromosome segregation by either complete absence of cytokinesis (Figure S3F) or transfer of the whole spindle to one daughter cell during the ongoing furrowing process (Figure 3C), similar to what we observed after PRC1 KO and EG5 inhibition (Figures 2C and S2N). In KIF4A-depleted cells, the blocked spindle elongation phenotype was also observed when EG5 was inhibited by using 100-μM monastrol (Figures 3A, 3B, S1A, and S3D) (Mayer et al., 1999), which unlike STLC does not inhibit microtubule-depolymerizing activity of EG5 (Kim et al., 2019), indicating that the EG5 role during anaphase B is mediated by its motility rather than microtubule-depolymerizing function. Interestingly, spindle elongation could be reactivated when STLC was washed out during early anaphase, suggesting that the block in spindle elongation is reversible (Figures S3G and S3H).

Video S4. Anaphase spindle elongation is abrogated after depletion of KIF4A by siRNA and inhibition (inh.) of EG5 by addition of STLC, related to Figure 3 Swept-field confocal time-lapse imaging of RPE-1 cells stably expressing CENP-A-GFP and centrin1-GFP under the indicated experimental conditions. Cells were recorded every 15 s. Time, s.

KIF4A accumulated in the spindle midzone during early anaphase following the accumulation of PRC1 and MKLP1 (Figures S3I and S3J) (Nunes Bastos et al., 2013; Kurasawa et al., 2004). Unlike KIF4A, individual depletions of MKLP1, MKLP2, CENP-E, or KIF14 combined with EG5 inhibition did not block spindle elongation (Figures 3B, S1A, S3A, S3D, and S3K), suggesting that the observed effects of their single depletions on spindle elongation velocities (Figures 1D, S1A, and S1B) were not modulated further by inhibition of EG5. Similarly, spindle elongation block was not induced when combining depletions of KIF18A or HSET with EG5 inhibition (Figures 3B, S1A, S1B, S3A, and S3D), although combining depletion of KIF18A with EG5 inhibition significantly slowed down spindle elongation with respect to control cells and KIF18A depletion alone (Figures 3B and S3A), implying possible redundancy with EG5 during anaphase spindle elongation.

### The kinesin-6 MKLP1 and kinesin-6 MKLP2 play a supporting role in spindle elongation

**Figure 4:**
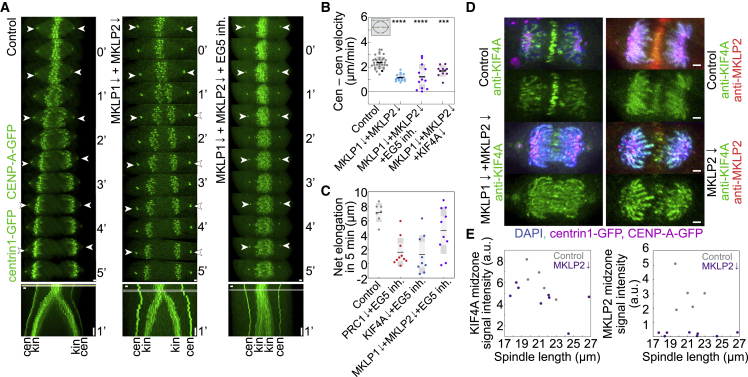
Depletion of both kinesin-6 MKLP1 and kinesin-6 MKLP2 with inactivation of EG5 induces a partial block in chromosome segregation by disrupting spindle elongation (A) Live-cell images of control, MKLP1- and MKLP2-siRNA co-depleted and MKLP1- and MKLP2 siRNA co-depleted 40-μM STLC-treated RPE-1 cells stably expressing CENP-A-GFP and centrin1-GFP. kin, kinetochore; cen, centrosome. White arrowheads indicate centrioles. Horizontal gray bars in the kymographs indicate the onset of anaphase. (B) Quantification of velocity of spindle elongation (n = 34, 17, 12, and 11 cells, from left to right). Three independent experiments for every condition regarding siRNAs or non-targeting treatments, while number of independent experiments regarding STLC treatment is equal to the number of cells. Black data dots in every treatment correspond to the measurements from the exemplar cells shown on the time-lapse images and kymographs (A). Statistics: t test (^∗^p < 0.05; ^∗∗^p < 0.01; ^∗∗∗^p < 0.001; ^∗∗∗∗^p < 0.0001). (C) Quantification of net spindle elongation from anaphase onset to 5-min afterward for treatments as indicated (n = 7, 10, 9, and 10 cells, from left to right). Time is shown as minutes. Images are the maximum projection of the acquired z stack. Time 0 represents anaphase onset. Horizontal scale bars, 1 μm. Vertical scale bars, 1 min. (D) Immunofluorescence images of fixed RPE-1 cells depleted of target proteins by treatment with indicated siRNAs stably expressing CENP-A-GFP and centrin1-GFP (magenta) stained with AlexaFluor594 conjugated with antibody specific to the indicated target protein (green KIF4A and red MKLP2). Chromosomes were stained with 1 μg/mL DAPI solution (cyan). Scale bars, 2 μm (D). (E) Quantification of mean signal intensity measured in the spindle midzone of KIF4A (top) and MKLP2 (bottom) antibodies conjugated with AlexaFluor594 versus spindle length in the indicated conditions (8 MKLP2 depleted and 5 control cells). Two independent experiments for every condition regarding siRNAs or non-targeting treatments. See also Figure S4.

Interestingly, double depletion of both kinesin-6s had a significant effect on spindle elongation, lowering the velocity more than 50% when compared with control cells (Figures 4A, 4B, S1A, S1D, and S4A–S4C), implying their role in the spindle elongation. Interestingly, co-depletion of MKLP1 and MKLP2 together with EG5 inhibition lowered spindle elongation velocities drastically compared with controls (spindle elongation velocity 0.89 ± 0.25 versus 2.36 ± 0.08 μm/min), inducing complete lack of spindle elongation in 30% of the cells (4 out of 12 cells; Figures 4A, 4B, S1A, S4A–S4D; Video S5), suggesting the effect of this double depletion can be modulated further by inhibition of EG5. The velocities of spindle elongation after the combined MKLP1, MKLP2, and EG5 perturbation were similar to those after the combined KIF4A and EG5 perturbations during the first 2 min of anaphase (Figure S1A). However, unlike in KIF4A/EG5 perturbation, the spindles in MKLP1/MKLP2/EG5 perturbation started to elongate 2 min after anaphase onset and increased their length by 4.34 ± 1.25 μm within 5 min from the onset, which is significantly more than the elongation of 1.23 ± 0.88 μm in KIF4A/EG5 and 1.50 ± 0.68 in PRC1/EG5 during the same time period, but less than 7.1 ± 0.51 μm in control cells (Figure 4C). These results suggest that KIF4A has a stronger effect on anaphase elongation than MKLP1 and MKLP2.

Video S5. Anaphase spindle elongation is abrogated after co-depletion of kinesin-6 MKLP1 and kinesin-6 MKLP2 by siRNA and inhibition (inh.) of EG5 by addition of STLC, related to Figure 4 Swept-field confocal time-lapse imaging of RPE-1 cells stably expressing CENP-A-GFP and centrin1-GFP under the indicated experimental conditions. Cells were recorded every 15 s. Time, s.

The role of MKLP1 and MKLP2 may be to regulate KIF4A activity in the midzone (Nunes Bastos et al., 2013). However, we observed that KIF4A is present on midzone microtubules after depletion of both MKLP1 and MKLP2 (Figures 4D, 4E, and S4E), suggesting that MKLP1 and MKLP2 do not regulate entirely the midzone localization of KIF4A, although MKLP2 regulates its microtubule residence time as shown by FRAP experiments on KIF4A-GFP in HeLa cells (Nunes Bastos et al., 2013). An alternative possibility is that KIF4A regulates MKLP1 and MKLP2. Yet, we found that MKLP1 localizes to midzone microtubules after KIF4A depletion (Figure S3I), arguing against this possibility. KIF4A depletion resulted in longer streaks of MKLP1 signal in the midzone (Figures S3I–S3J), which mimicked longer PRC1 streaks (Figures S3I–S3J), in agreement with previous studies (Zhu and Jiang, 2005). Also, KIF4A was present on the midzone microtubules after efficient depletion of HSET (Figure S4F), contrary to situation observed in HeLa cells where HSET depletion induced defects in localization of the midzone components (Cai et al., 2010).

We hypothesize that KIF4A regulation probably involves Aurora B kinase (Nunes Bastos et al., 2013; Douglas et al., 2010; Ozlü et al., 2010; Gruneberg et al., 2004), whose inhibition by both Barasertib and ZM 447439 inhibitors (Afonso et al., 2014; Yang et al., 2007) also slowed down spindle elongation but the velocity of elongation was not decreased further by combined EG5 inhibition by STLC (Figures S1A, S4A, S4B, and S4G) similarly to EG5 inhibition by FCPT (Afonso et al., 2014), implicating more complex mechanisms. Finally, after triple-combined depletion of both kinesin-6s and KIF4A, we did not observe functional redundancy of target proteins in regard of the impact on spindle elongation velocities when compared with double depletion of kinesin-6s and their individual depletions (Figures 4B, S1A, and S4A–S4C). Taken together, these results suggest that together with EG5, KIF4A is the main motor driving microtubule sliding, while MKLP1 and MKLP2 play a secondary role (Figure S4H).

### Increase in the stability of midzone microtubules is not essential for spindle elongation during early anaphase

**Figure 5:**
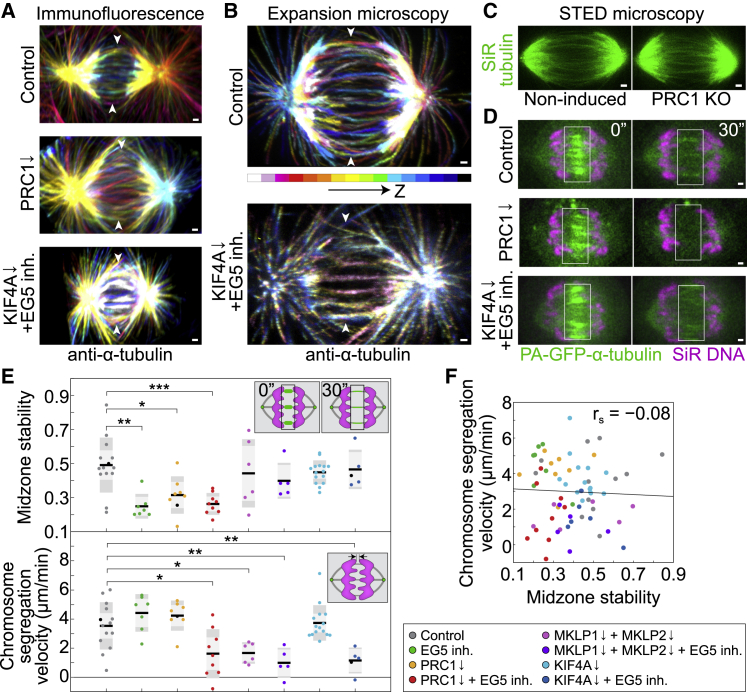
Decreased midzone stability does not affect chromosome segregation velocity during anaphase (A and B) Immunofluorescent (A) and expansion microscopy (B) images of fixed control, PRC1-siRNA-depleted (only immunofluorescence) and KIF4A-siRNA-depleted 40-μM STLC-treated RPE-1 cells stained with AlexaFluor594 conjugated with α-tubulin antibody. Images in (A) and (B) are the maximum projections of color-coded z stacks as shown by the scheme in (B). Arrowheads point to the spindle midzone region. (C) STED images (single z plane) of anaphase spindles labeled with 100 nM SiR-tubulin in live non-induced CRISPR PRC1 KO and induced PRC1 KO RPE-1 cells. (D) Smoothed live-cell images (single z plane) of RPE-1 cells after midzone photoactivation of photoactivatable (PA)-GFP-α-tubulin (green) in the indicated conditions. 100-nM SiR-DNA (magenta) was used for chromosome staining. Time 0 represents the photoactivation onset. (E) Quantification of midzone stability, defined as the ratio of the integrated photoactivated signal in the boxed region (see schemes and boxes in D) at times 30 and 0 s, and chromosome segregation velocity measured in the same time period (see schemes), for the indicated treatments. Statistics: t test (^∗^p < 0.05; ^∗∗^p < 0.01; ^∗∗∗^p < 0.001; ^∗∗∗∗^p < 0.0001). Numbers: 14, 7, 8, 9, 6, 5, 15, and 5 cells, from left to right. Three independent experiments for every condition except MKLP1 + MKLP2, MKLP1 + MKLP2 + EG5 inh. and KIF4+EG5 inh., which were done in two independent experiments regarding siRNAs or non-targeting treatments, while number of independent experiments regarding STLC treatment is equal to the number of cells. Black data dots in every treatment correspond to the measurements from the exemplar cells shown on the time-lapse images and kymographs (D). (F) Chromosome segregation velocity versus midzone stability (data from E) for indicated treatments and linear regression (line); r_s_, Spearman correlation coefficient, p < 0.001. Scale bars, 1 μm. See also Figure S5.

KIF4A and EG5 may promote spindle elongation passively by organizing the midzone architecture and/or actively by generating forces that drive the elongation. To investigate the first possibility, we immunolabeled α-tubulin using a microtubule-preserving protocol (see STAR Methods) and showed that midzone microtubules were present and organized as discrete bundled structures across different treatments, including those that blocked spindle elongation (Figures 5A and S5A). We measured integrated intensity of tubulin within the midzone after KIF4A depletion and acute EG5 inhibition and found no significant difference when compared with control untreated cells (Figure S5B). Similar results regarding organized and discrete midzones after KIF4A depletion and EG5 inhibition were obtained by using super-resolution expansion microscopy (Chen et al., 2015) on α-tubulin-immunolabeled specimens (Figures 5B, S5C, and S5D), suggesting that the observed chromosome segregation block was not due to total loss of the organization of the midzone microtubules. Interestingly, PRC1 depletion by siRNA or KO by CRISPR did not result in highly disorganized midzones during early anaphase, as shown both by immunofluorescence (Figures 5A and S5A) and stimulated emission depletion (STED) microscopy (Vicidomini et al., 2018) (Figures 5C and S5E), contrary to what is observed in late anaphase spindles (Pamula et al., 2019; Mollinari et al., 2002). However, tubulin signal intensities after PRC1 KO were slightly decreased and microtubule bundles were less defined when compared with the non-induced control cells (Figures 5C and S5E), suggesting PRC1 is poorly involved in midzone organization during early anaphase and this function of PRC1 is mostly restricted to late anaphase/telophase events in human cells.

Given that depletions of PRC1 or MKLP1 decrease midzone microtubule stability (Vukušić et al., 2017; Mollinari et al., 2002) and over-bundling of midzone microtubules upon overexpression of PRC1 slowed down spindle elongation (Figures S5F and S5G), the blocked spindle elongation may be due to disrupted microtubule stability. By using fluorescence dissipation after photoactivation (FDAPA) of photoactivatable (PA)-GFP-α-tubulin within the spindle midzone (Figures 5D and S5H), we found that microtubule stability was reduced in treatments including depletion or KO of PRC1 (Figures 5E and S5H; Video S6). Surprisingly, chromosome segregation and spindle elongation velocities were not correlated with microtubule stability (Figure 5F), demonstrating that the blocked spindle elongation induced by perturbations of kinesins and PRC1 does not depend on microtubule turnover within the midzone. Thus, the increased midzone microtubule stability observed in late anaphase, which results primarily from the microtubule bundling activity of PRC1 (Mollinari et al., 2002), is not required for proper spindle elongation during early anaphase, arguing against a solely passive microtubule-organizing role of KIF4A and EG5.

Video S6. Reduction of microtubule stability seen after PRC1 depletion does not induce impairment of chromosome segregation, related to Figure 5 Swept-field confocal time-lapse imaging of RPE-1 cells stably expressing PA-GFP-α-tubulin (green) labeled with 100 nM SiR-DNA (magenta) under the indicated experimental conditions, after photoactivation of tubulin with 405-nm laser. Cells were recorded every 0.8 s. Time, s.

### Astral microtubules are not crucial for spindle elongation

In conditions that blocked spindle elongation, including depletion of KIF4A and inhibition of EG5, astral microtubules were observed close to the cell boundary, similar to control cells (Figure S5A). The length of astral microtubules in mid-anaphase spindles measured by using immunolabeled α-tubulin was not different between control cells and KIF4A depleted cells treated with STLC for 5 min (Figure S5I). This observation argues against disruption of astral microtubules in those conditions, suggesting that the spindle elongation was not blocked due to disruption of cortical pulling forces.

**Figure 6:**
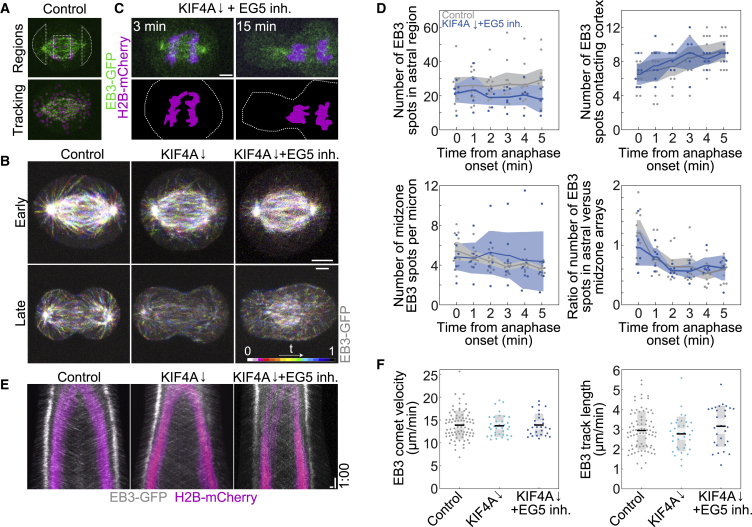
KIF4A depletion and EG5 inhibition does not impact the number or dynamic properties of midzone and astral microtubules (A) Live-cell images of control RPE-1 cells expressing EB3-eGFP and H2B-mCherry showing schematically the definition of two astral regions, and the midzone region between H2B-labeled chromosomes (top) and the same cell with tracked EB3 spots (magenta circles) using TrackMate ImageJ tool (bottom). (B) Live-cell images of RPE-1 cells expressing EB3-eGFP in control, KIF4A siRNA depletion and KIF4A siRNA depletion and 40-μM STLC treatment in early anaphase (top row) and late anaphase (bottom row). Images are maximum temporal projections of color-coded time points as shown on the scheme generated from a total of 1 min of early and late anaphase time frames. (C) Live-cell images (top) and corresponding schemes (bottom) of RPE-1 cells in mid anaphase (3 min from the onset) and telophase (15 min from the onset) after KIF4A depletion and EG5 inhibition. Dashed lines indicate the cell borders. (D) Graphs depicting change in the number of EB3 spots in astral regions (first), number of EB3 spots contacting the cortical area (second), number of EB3 spots per micron of midzone (third), and ratio of the number of EB3 spots in astral versus midzone arrays (fourth) in time in both control (n = 12 cells from two independent experiments) and KIF4A-depleted STLC-treated spindles (n = 6 cells from two independent experiments). Shaded areas represent the 95% confidence interval of the mean and thick lines represent the mean values for both indicated treatments. (E) Whole spindle kymographs of EB3-GFP (gray) and H2B-mCherry-expressing (magenta) cells during 10 min of anaphase in the indicated treatments. (F) Quantification of EB3 comet velocity (left) and EB3 comet track length (right) in the indicated treatments (n = 3 cells for KIF4A depletion from two independent experiments, other treatments are the same as in D; the number of comet traces is 80, 35, and 28 from left to right). Statistics: t test. Horizontal scale bars, 5 μm (in E, 1 μm). Vertical scale bar, 1 min. See also Figure S6.

To study the effect of KIF4A depletion and EG5 inhibition on dynamical properties of astral and other dynamic microtubules, we used RPE-1 cell line with GFP-labeled plus-end-tracking protein EB3 (Figure 6A) (Akhmanova and Steinmetz, 2008), and chromosome marker H2B labeled with mCherry. After KIF4A depletion and EG5 inhibition, we observed negligible spindle elongation when compared with both KIF4A-depleted and control cells (Figure 6B), while in all cells growing EB3 spots were observed in both early and late anaphase spindles (Figure 6B), and integrated fluorescence intensity of EB3 during first minute of anaphase did not differ between control and KIF4A-depleted EG5-inhibited spindles (Figure S6A). Furthermore, combined KIF4A depletion and EG5 inhibition in half of the imaged cells (3 out of 6 cells) resulted in the whole chromosome mass positioned in one daughter cell after cytokinesis (Figure 6C), similar to our observations after KIF4A depletion and EG5 inhibition in another cell line (Figure 3C).

To explore whether astral microtubules were perturbed by KIF4A depletion and EG5 inhibition by STLC, we compared the total number of EB3-GFP spots in the astral arrays (Figures 6A and 6D) and the number of EB3-GFP spots contacting the cortical area during multiple time points of anaphase and found no significant difference between control and KIF4A-depleted, STLC-treated spindles (Figure 6D). The total number of EB3 spots per micron of the midzone array (Figures 6A, 6D, and S6B) did not change after depletion of KIF4A and inhibition of EG5 when compared with control cells (Figure 6D). Finally, the ratio of spots in the astral and midzone arrays decreased in a similar manner through anaphase in control and KIF4A-depleted, STLC-treated spindles (Figure 6D). Taken together, these results suggest that KIF4A and EG5 perturbation does not impact the number of growing astral and central spindle microtubules during anaphase spindle elongation.

To investigate the effect of KIF4A and EG5 perturbation on microtubule polymerization, we quantified the velocity and stability of EB3 comets during early anaphase in the whole spindle and found that KIF4A depletion, with or without STLC treatment, did not change the comet velocity nor its track length when compared with control spindles (Figures 6E, 6F, and S6C). These results suggest that KIF4A and EG5 do not affect growth of microtubule plus ends in the whole spindle during early anaphase. Thus, we conclude that KIF4A and EG5 do not influence the net polymerization of microtubules during early anaphase and that they do not affect the number of growing midzone or astral microtubules nor the number of astral microtubules reaching the cortical area of the cell during anaphase.

### KIF4A and EG5 slide midzone microtubules apart

**Figure 7:**
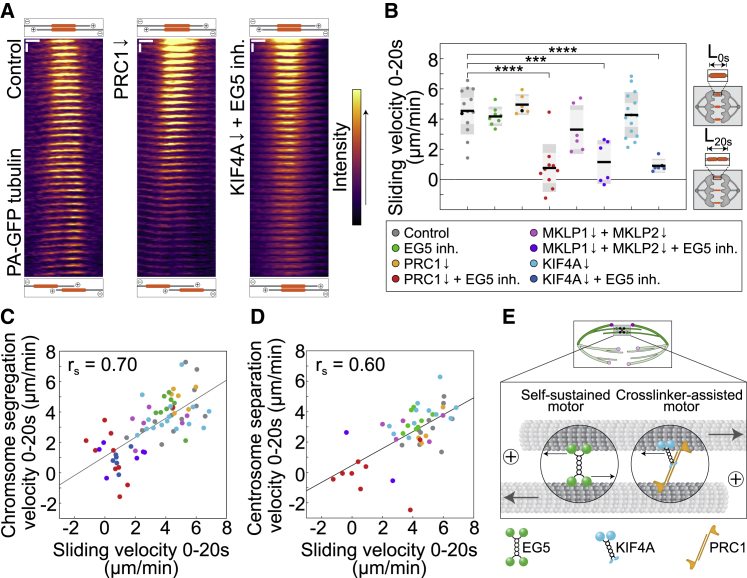
Chromosome segregation and spindle elongation are dependent upon KIF4A and EG5-generated sliding (A) Montage time-lapse live images of the mitotic spindle midzone region after photoactivation of photoactivatable (PA)-GFP-α-tubulin in the indicated treatments. Top and bottom schemes depict sliding of the photoactivated midzone region. Vertical scale bar, 1 s. Horizontal scale bar, 1 μm. (B) Quantification of sliding velocity, measured as the length change of the photoactivated spot (L, see scheme) in the indicated conditions. Statistics: t test (^∗^p < 0.05; ^∗∗^p < 0.01; ^∗∗∗^p < 0.001; ^∗∗∗∗^p < 0.0001). Numbers: 13, 8, 6, 10, 6, 6, 15, and 6 cells, from left to right. Three independent experiments for every condition except MKLP1 + MKLP2 siRNAs and MKLP1 + MKLP2 + EG5 inh., which were done in two independent experiments regarding siRNAs or non-targeting treatments, while number of independent experiments regarding STLC treatment is equal to the number of cells. Black data dots in every treatment correspond to the measurements from the exemplar cells shown on the time-lapse images and kymographs (A and B). (C and D) Chromosome segregation velocity (C) and centrosome separation velocity (D) versus sliding velocity in the indicated treatments, and linear regression (line); r_s_, Spearman correlation coefficient, p < 0.001. (E) Proposed model for the motor-driven sliding in the antiparallel midzone region (top scheme) of an anaphase mitotic spindle including two independent sliding modules. Gray arrows point to the direction of overall microtubule motion as a result of forces produced by the motors walking on antiparallel microtubules (black arrows). See also Figure S7.

Finally, to test whether KIF4A and EG5 drive sliding of antiparallel microtubules, we measured sliding of midzone microtubules after photoactivation of PA-GFP-α-tubulin (Figure S7A). The sliding velocity was significantly reduced after simultaneous depletion of KIF4A and EG5 inhibition compared with controls (Figures 7A, 7B, and S7A–S7C; Video S7). In line with these data, all other conditions that impact KIF4A activity, when combined with EG5 inhibition, mimicked this phenotype of reduced sliding velocities (Figures 7B and S7A–S7C). Moreover, chromosome segregation and spindle elongation velocities strongly correlated with sliding rates across conditions (Figures 7C, 7D, S7D, and S7E), which suggests that the origin of blocked spindle elongation seen after perturbations of KIF4A and EG5 is a result of a defective microtubule sliding. Given that human KIF4A and EG5 promote microtubule depolymerization (Kim et al., 2019; Bringmann et al., 2004), it is unlikely that these proteins drive spindle elongation via microtubule polymerization forces (Figure 1A, right) because their depletion is expected to promote spindle elongation rather than spindle elongation block as observed here. We conclude that KIF4A and EG5 drive anaphase spindle elongation together by moving along the antiparallel microtubules in the midzone to push them apart (Figures 1A left and 7E).

Video S7. Sliding of midzone microtubules is greatly perturbed after depletion of KIF4A by siRNA and inhibition (inh.) of EG5 by addition of STLC, related to Figure 7 Swept-field confocal time-lapse imaging of RPE-1 cells stably expressing PA-GFP-α-tubulin (green) labeled with 100 nM SiR-DNA (magenta) under the indicated experimental conditions, after photoactivation of tubulin with 405-nm laser. Top cell is recorded at 1 s and the rest at 0.8 s. The top video is played at 11.3 fps, and the rest at 14 fps, which is 2 times faster than recorded. The fps rate was adjusted in a way that all movies can end up simu ltaneously. Time, s.

## Discussion

### Two redundant protein modules are crucial for anaphase spindle elongation

Our work reveals the long-sought mechanism of anaphase spindle elongation in human cells. We identified EG5 and PRC1 proteins as parts of distinct crucial biochemical modules required for anaphase B in human cells (Vukušić and Tolić, 2021). Our work demonstrates the role of EG5 in anaphase spindle elongation in mammalian cells, a concept that has been under debate for decades (Civelekoglu-Scholey and Scholey, 2010; Kapitein et al., 2005). EG5 is a plus-end-directed motor capable of sliding microtubules *in vitro* (Kapitein et al., 2005; Kashina et al., 1996) and is crucial for maintenance of spindle length during metaphase in human non-transformed cells (Gayek and Ohi, 2014; van Heesbeen et al., 2014). On the other hand, EG5 alone seems to be dispensable for anaphase spindle elongation in human cells after inhibition with STLC, which induces its weak-binding state to microtubules (Skoufias et al., 2006), and similar findings were also reported in *Drosophila* embryo mitosis after injection of antibodies against kinesin-5 (Brust-Mascher et al., 2009). Moreover, the lack of effect on spindle elongation after EG5 inhibition cannot be explained by activity of another plus-end-directed motor, KIF15, which acts redundantly with EG5 in control of spindle length during metaphase in human tumor cell lines (van Heesbeen et al., 2014; Tanenbaum et al., 2009). Contrary to EG5 inhibition with STLC, inhibition with FCPT, which locks EG5 in a rigor-bound state to microtubules (Groen et al., 2008), induces drastic perturbation of spindle elongation (Afonso et al., 2014), probably by inducing large frictional forces in the midzone. Our results in which PRC1 is depleted concurrently with EG5 inhibition support the conclusion that the protein, which is capable of supporting spindle elongation in the absence of EG5 activity, is PRC1-dependent. PRC1 is a passive crosslinker of antiparallel microtubules (Mollinari et al., 2002), and it is unlikely that it drives spindle elongation directly, but on the other hand, PRC1 is important for the organization of the spindle midzone and for recruitment of numerous force-generating and regulatory proteins specifically to the spindle midzone upon anaphase onset (Lee et al., 2012; Kurasawa et al., 2004).

Although PRC1 is important for the increased midzone microtubule bundling and stability in the central spindle during late anaphase (Pamula et al., 2019), a function mediated by a decrease in PRC1 phosphorylation after anaphase onset (Hu et al., 2012), it remained unclear if this increase in the microtubule bundling and stability is important for all stages during anaphase. Our results suggest that the increased midzone microtubule stability and bundling are not crucial for spindle elongation during early anaphase. However, bundling may be important for later stages of anaphase, where the increased accumulation of PRC1 could induce frictional forces that oppose further sliding in the midzone (Gaska et al., 2020; Lansky et al., 2015). Our experiments in which PRC1 overexpression slowed down spindle elongation in early anaphase support this idea. Similarly, PRC1 perturbation impacts significantly the structure of late anaphase spindles (Pamula et al., 2019), while the effect on early and mid-anaphase spindles is not so apparent, suggesting that the role of PRC1 in microtubule bundling is upregulated during the late anaphase, a notion supported by recent FRAP experiments on PRC1 (Asthana et al., 2020).

On the other hand, our data show that the scaffolding function of PRC1 is more important for spindle elongation during early anaphase because perturbation of its downstream partners, KIF4A or both kinesin-6s simultaneously combined with EG5 inhibition, can mimic the effect seen after perturbation of PRC1 and inhibition of EG5. Among PRC1-interacting motors, KIF4A completely mimics the blocked spindle elongation phenotype seen after PRC1 perturbation and EG5 inhibition, suggesting that the necessity of PRC1 for spindle elongation is due to recruitment of KIF4A. Interestingly, a recent *in vitro* study proposed that the interaction with PRC1 is needed for antiparallel sliding of KIF4A in the microtubule overlap (Wijeratne and Subramanian, 2018). Our experiments in which spindle elongation is completely blocked by perturbation of PRC1 combined with EG5 inhibition support this view, implicating PRC1-KIF4A interaction as crucial for microtubule-sliding activity of KIF4A in human cells. Interestingly, a similar redundancy between EG5-KIF4A-KIF15 motors has been observed recently in the regulation of microtubule poleward flux during metaphase in human tumor cell lines (Steblyanko et al., 2020).

### Antiparallel microtubule sliding by KIF4A and EG5 drive spindle elongation

In contrast to their individual depletions, microtubule sliding is abrogated in all conditions where KIF4A localization or activity is perturbed together with EG5 motor activity. This indicates that KIF4A and EG5 exert forces during anaphase by sliding apart antiparallel microtubules. Both motors are capable of sliding microtubules *in vitro* (Wijeratne and Subramanian, 2018; Kapitein et al., 2005). In our model (Figure 7E), KIF4A acts as a microtubule-sliding protein in the spindle, which is a new function of KIF4A, given that so far this motor has mainly been implicated in midzone length regulation during late anaphase (Hu et al., 2011) due to its plus-end polymerization inhibiting activity (Bringmann et al., 2004). Thus, our work provides a missing link that demonstrates the ”sliding filament mechanism” (McIntosh et al., 1969) during anaphase by identifying two key protein modules involved in antiparallel microtubule sliding, one based on the EG5 motor, which generates outward midzone forces throughout cell division (Gable et al., 2012; Blangy et al., 1995), and the other where the main player is KIF4A, which turns on after anaphase onset (Kurasawa et al., 2004) (Figure 7E). Interestingly, two sliding modules differ in the mechanism of sliding; kinesin-5 generates force as a self-sustained homotetrameric bipolar sliding protein (Kapitein et al., 2005; Kashina et al., 1996), whereas kinesin-4 is a homodimer, which requires interaction with the crosslinker PRC1 to perform efficient sliding (Wijeratne and Subramanian, 2018). Sliding forces generated in the midzone by these modules are then transmitted to the spindle poles through lateral connections to k-fibers, which were shown to be an important part of anaphase segregation machinery (Vukušić et al., 2017).

After anaphase onset, chromosomally localized KIF4A starts to accumulate on the midzone microtubules due to its interaction with PRC1 (Kurasawa et al., 2004), and we propose that its activity is regulated by a cascade involving kinesin-6s and Aurora B (Nunes Bastos et al., 2013; Gruneberg et al., 2004) (Figure S4H) because our results show that perturbations of those proteins can partially mimic the phenotype of KIF4A depletion and EG5 inhibition. Aurora B regulates KIF4A by direct phosphorylation (Nunes Bastos et al., 2013), thereby promoting its association with microtubules. However, our experiments indicate that KIF4A can localize on midzone microtubules even after reduction of MKLP2 levels, yet previous work found highly reduced KIF4A-GFP within the midzone after MKLP2 depletion in HeLa cells (Nunes Bastos et al., 2013). This discrepancy could be attributed to different efficiency of MKLP2 knockdown or different cell lines. Moreover, we find it unlikely that spindle elongation is driven by direct sliding of kinesin-6s because their combined depletion does not slow down microtubule sliding. As depletion of both kinesin-6s combined with EG5 inhibition does not mimic the depletion of PRC1, contrary to KIF4A, they could be involved in the spindle elongation via a PRC1-independent pathway, possibly through the transport function of MKLP2 that is essential for the localization of chromosomal passenger complex to the midzone (Adriaans et al., 2020). If true, this suggests that the observed lower velocities during early anaphase after the depletion of kinesin-6s might result from the induced delay in the start of the anaphase B possibly by controlling the loading of sliding motors such as KIF4A (Nunes Bastos et al., 2013), or their regulators, such as Aurora B (Gruneberg et al., 2004). Alternatively, KIF4A could be crucial for the regulation of MKLP1 and MKLP2, either through regulation of their localization or activity within the midzone. We do not favor this scenario because our experiments show that after KIF4A depletion MKLP1 still localizes to the spindle midzone, although in a broader pattern compared with the control cells, similar to PRC1 and its other interacting partners such as CENP-E and Aurora B (Hu et al., 2011; Kurasawa et al., 2004).

Our observation that the effect of KIF18A depletion on spindle elongation velocity could be modulated further by EG5 inhibition indicates that EG5 and KIF18A could form a functionally redundant partnership during anaphase B, although not to the same extent as KIF4A and EG5, as spindle elongation was not blocked after combined KIF18A-EG5 perturbations. KIF18A was recently reported to localize to metaphase bridging microtubules (Jagrić et al., 2021), and we also observed clear localization of KIF18A to the spindle midzone from anaphase onset, similar to a previous study (Stumpff et al., 2012). Kinesin-8 Kip3 has been reported to slide antiparallel microtubules during mitosis in the budding yeast (Su et al., 2013), indicating a possible similar role of the human homolog KIF18A that could be an interesting topic of future studies. In addition, from our results HSET does not seem to play an important role in midzone organization during anaphase in RPE-1 cells, and consequently, localization of midzone components like KIF4A, which was reported previously for HeLa cells (Cai et al., 2010). This is in agreement with a lack of effect of HSET depletion on the global increase in spindle multipolarity in RPE-1 cells when compared with the same depletion in human transformed cell lines (Kleylein-Sohn et al., 2012), indicating possible differences in the HSET activity.

We argue that direct pushing by plus-end polymerization is excluded as a major force driving spindle elongation because KIF4A and EG5 promote microtubule depolymerization (Kim et al., 2019; Bringmann et al., 2004); thus, in their absence microtubules are expected to polymerize more, although this was not found in our experiments, and promote spindle elongation as reported during late anaphase (Hu et al., 2011), which is the opposite from the block of spindle elongation we observed. Similarly, cortical forces exerted by dynein on astral microtubules most likely do not drive spindle elongation in early anaphase, based on our results that astral microtubules were not perturbed when spindle elongation was blocked, and on the fact that the role of dynein in chromosome segregation is restricted to late anaphase events, as shown by perturbation of specific anaphase cortical adaptors of dynein (Kiyomitsu and Cheeseman, 2013).

### Impact of defects in spindle elongation on chromosome segregation fidelity

Interestingly, we observed complete failure of chromosome segregation due to lack of spindle elongation. The observed defects most likely result in cell tetraploidization after completion of cell division through three distinct pathways: (1) complete absence of cytokinesis, (2) cytokinesis failure due to chromatin mass trapped in the active cleavage plane (Steigemann et al., 2009; Weaver et al., 2006), or (3) complete relocation of the chromosome mass to one daughter cell during ongoing cleavage process. The absence of cytokinesis could be due to lack of activity of polo-like kinase-1 (PLK1) in the spindle midzone, as observed previously (Brennan et al., 2007; Petronczki et al., 2007), which may be a consequence of the absence of spindle elongation in our experiments. Previous studies also reported that cytokinesis failure due to trapped chromatin in the cleavage plane is the major cause for spontaneous tetraploidization in cultured cells that has a huge oncogenic potential, which often leads to stable aneuploidy phenotype frequently observed in human tumors (Lens and Medema, 2019; Fujiwara et al., 2005).

In conclusion, our results show that mechanistically distinct motor modules, based on KIF4A-MKLP1-MKLP2 molecular motors on one side and EG5 on the other, work together to perform the same function in anaphase spindle elongation, a concept which may be widespread across motor-driven processes from mitotic and meiotic spindle assembly to viral trafficking and axonal transport.

### Limitations of the study

This work proposes a model of anaphase B in human cells driven by the combined action of EG5 and KIF4A motors sliding antiparallel midzone microtubules. However, MKLP1, MKLP2, and KIF18A also showed redundant activity with EG5, although to a lesser extent compared with KIF4A. It is possible that MKLP1 and MKLP2 are involved in the regulation of KIF4A localization or activity. Alternatively, these motors may be part of a new redundant module driving spindle elongation independently of KIF4A, which will be an interesting topic for future studies. Furthermore, clearer discrimination between the role of KIF4A in the regulation of plus-end dynamics of midzone microtubules and its role in MT sliding awaits more experimental data. Finally, due to the use of siRNA-mediated knockdown to reduce protein levels within the cell, variable knockdown efficiency of target genes could interfere with some interpretations.

## STAR★Methods

### Key resources table

**Table:** 

REAGENT or RESOURCE	SOURCE	IDENTIFIER
**Antibodies**		

mouse monoclonal PRC1	Santa Cruz Biotechnology	Cat# sc-376983
mouse monoclonal EG5	Santa Cruz Biotechnology	Cat# sc-365681; RRID: AB_10841907
mouse monoclonal KIF4A	Santa Cruz Biotechnology	E-8, Cat# sc-365144; RRID: AB_10707683
rabbit polyclonal MKLP-1	Santa Cruz Biotechnology	Cat# sc-867; RRID: AB_631959
rabbit polyclonal KIF20A	Bethyl Laboratories	Cat# A300-879A; RRID: AB_2131560
rat anti-alpha Tubulin YL1/2	Invitrogen	Cat# MA1-80017; RRID: AB_2210201
rabbit polyclonal KIF14	Abcam	Cat# ab3746; RRID: AB_304043
mouse monoclonal KIFC1	Santa Cruz Biotechnology	M-63, Cat# sc-100947; RRID: AB_2132540
rabbit polyclonal KIF18A	Bethyl Laboratories	Cat# A301-080A; RRID: AB_2296551
rabbit polyclonal KIF4A	Bethyl Laboratories	Cat# A301-074A; RRID: AB_2280904
donkey anti-mouse IgG Alexa Fluor 594	Abcam	Cat# ab150112; RRID: AB_2813898
donkey anti-rabbit IgG Alexa Fluor 594	Abcam	Cat# ab150064; RRID: AB_2734146
donkey anti-rabbit IgG Alexa Fluor 647	Abcam	Cat# ab150075; RRID: AB_2752244
donkey anti-mouse IgG Alexa Fluor 647	Abcam	Cat# ab150107; RRID: AB_2890037
donkey anti-rat IgG Alexa Fluor 594	Abcam	Cat# ab150156; RRID: AB_2890252
mouse anti-KIF20A	Santa Cruz Biotechnology	D-3, Cat# sc-374508; RRID: AB_10989938
mouse anti-GAPDH	Abcam	Cat# ab9485; RRID: AB_307275
mouse anti-beta-actin	Invitrogen	Cat# PA1-183; RRID: AB_2539914
HRP-conjugated goat anti-mouse secondary antibodies	Abcam	Cat# ab205719; AB_2755049

**Chemicals, Peptides, and Recombinant Proteins**		

(+)-S-Trityl-L-cysteine	Sigma-Aldrich	164739-5G
GSK-923295	MedChemExpress	HY-10299
ZM 447439	Selleckchem	S1103
Barasertib (AZD1152-HQPA)	Selleckchem	S1147
Monastrol	Sigma-Aldrich	M8515
Trypsin/EDTA	Biochrom AG	**N/A**
6-((Acryloyl)amino)hexanoic Acid, Succinimidyl ester (Acryloyl-X)	Thermo Fisher Scientific	A-20770
NaCl	Sigma-Aldrich	S7653
KCl	Sigma-Aldrich	P9333
Dimethyl sulfoxide	Thermo Fisher Scientific	D12345
MgCl_2_	Sigma Aldrich	M8266
Borohydride	Sigma Aldrich	213462
Sodium acrylate	Sigma Aldrich	408220
PIPES	Sigma Aldrich	P6757-500G
EDTA	Sigma Aldrich	EDS
Triton-X-100	Sigma Aldrich	93426
Paraformaldehyde 4%	Santa Cruz Biotechnology	sc-281692
Glutaraldehyde 50%	Sigma-Aldrich	G7651
Phosphate-buffered saline	Dulbecco	L 182-50
Immunopure Normal Goat Serum (iNGS)	Thermo-Fisher Scientific	31872
Silicone rhodamine (SiR)-DNA	Spirochrome AG	sc007
40% Acrylamide/Bis-acrylamide solution 29:1	Bio-Rad	161– 0146
Ammonium persulfate (APS)	Bio-Rad	161– 0700
N, N, N_0_, N_0_-tetramethylethane-1,2-diamine (TEMED)	Sigma-Aldrich	T7024
Tris(hydroxymethyl)aminomethane (TRIS)	Carl Roth GmbH + Co. KG	4855.2
Guanidine-HCl	Sigma-Aldrich	G45059
Proteinase-K	Sigma-Aldrich	P4850
SiR-tubulin	Spirochrome AG	SC002
RIPA buffer	Sigma Aldrich	R0287
1x protease inhibitor	Roche	5892970001
1x phosphatase inhibitor	Roche	4906837001
Tween 20	Sigma Aldrich	9005-64-5
100% milk	Bebimil	N/A
WesternSure PREMIUM Chemiluminescent Substrate	LI-COR	926-95000

**Critical Commercial Assays**		

Lipofectamine RNAiMAX Reagent	Invitrogen	13778-150
Amaxa Cell Line Nucleofactor Kit R	Lonza	VCA-1001
MycoAlert Mycoplasma Detection Kit	Lonza	LT07-118

**Experimental Models: Cell Lines**		

U2OS cell lines (human osteosarcoma, female), wild type and permanently transfected and stabilized using CENP-A-GFP	Laboratory of Helder Maiato, Institute for Molecular Cell Biology, University of Porto, Portugal (Barisic et al., 2015)	N/A
human hTERT-RPE-1 (retinal pigmented epithelium, female) permanently transfected and stabilized using CENP-A-GFP and centrin1-GFP	Laboratory of Alexey Khodjakov, Wadsworth Center, New York State Department of Health, Albany, NY (Magidson et al., 2011)	N/A
human hTERT-RPE-1 permanently transfected and stabilized using PA-GFP-α-tubulin	Laboratory of Patrick Meraldi, Faculty of Medicine, University of Geneva, Switzerland (Jaqaman et al., 2010)	N/A
human hTERT-RPE-1 permanently transfected and stabilized using H2B-mCherry and EB3-eGFP	Laboratory of Patrick Meraldi, Faculty of Medicine, University of Geneva, Switzerland	N/A
human hTERT-RPE-1 inducible CRISPR/Cas9 PRC1-G2.2 knock-out	Laboratory of Iain Cheeseman, Massachusetts Institute of Technology, Cambridge, MA, USA (McKinley and Cheeseman, 2017)	http://cellcycleknockouts.wi.mit.edu/
human HeLa cell lines (human adenocarcinoma, female) separately permanently transfected and stabilized using BAC containing EG5-GFP, MKLP1-GFP, KIF4A-GFP and PRC1-GFP	Laboratory of Tony Hyman, Max Planck Institute of Molecular Cell Biology and Genetics, Dresden, Germany (Poser et al., 2008)	N/A

**Oligonucleotides**		

human MKLP1 siRNA	Santa Cruz Biotechnology	sc-35936
human KIF14 siRNA	Santa Cruz Biotechnology	sc-60882
human KIF4A siRNA	Santa Cruz Biotechnology	sc-60888
human KIF20A	Santa Cruz Biotechnology	sc-91657
human KIFC1 siRNA	Santa Cruz Biotechnology	sc-95157
control siRNA	Santa Cruz Biotechnology	sc-37007
human ON-TARGET KIF15 siRNA	Dharmacon	L-004960-00-0010
human ON-TARGET PRC1 siRNA	Dharmacon	L-019491-00-0020
control siRNA	Dharmacon	D001810-10-05
human Silencer Select Validated Kif18A siRNA	Ambion	s37882

**Recombinant DNA**		

PRC1-mCherry plasmid	Laboratory of Casper C. Hoogenraad, Utrecht University, Utrecht, Netherland	N/A
mRFP-CENP-B	Laboratory of Linda Wordeman, University of Washington, Seattle, WA, USA	N/A

**Software and Algorithms**		

ImageJ/Fiji	Schneider et.al., 2012	https://imagej.nih.gov/ij/
Matlab	The Mathworks, Inc.	https://nl.mathworks.com/products/matlab.html
SciDavis	Free Software Foundation, Inc., Boston, MA, USA	http://scidavis.sourceforge.net/
Adobe Illustrator CS6 and Adobe Photoshop CS6	Adobe Systems, Mountain View, CA, USA	https://www.adobe.com/

### Resource availability

#### Lead contact

Further information and requests for resources should be directed to and will be fulfilled by the Lead Contact, Iva M. Tolić (tolic@irb.hr).

#### Materials availability

All unique reagents generated in this study are available from the Lead Contact without restriction.

#### Data and code availability

The datasets and codes generated in this study will be made available on request from the Lead contact without restrictions.

### Experimental model and subject details

#### Cell lines

The human cell lines used are: **1.** human U2OS cell lines (human osteosarcoma, female) both wild type and permanently transfected and stabilized using CENP-A-GFP (protein of kinetochore complex), were a gift from Marin Barišić and Helder Maiato (Institute for Molecular Cell Biology, University of Porto, Portugal) (developed in Barisic et al., 2015), **2.** human hTERT-RPE-1 (retinal pigmented epithelium, female) permanently transfected and stabilized using CENP-A-GFP and centrin1-GFP (protein of a centrosome complex), which was a gift from Alexey Khodjakov (Wadsworth Center, New York State Department of Health, Albany, NY) (developed in Magidson et al., 2011), **3.** human hTERT-RPE-1 permanently transfected and stabilized using PA-GFP-α-tubulin and human hTERT-RPE-1 permanently transfected and stabilized using H2B-mCherry and EB3-eGFP which were a gift from Patrick Meraldi (Faculty of Medicine, University of Geneva, Switzerland) (developed in Jaqaman et al., 2010), **4.** human hTERT-RPE-1 inducible CRISPR/Cas9/PRC1-G2.2 knock-out (KO) which was a gift from Iain Cheeseman (Massachusetts Institute of Technology, Cambridge, MA, USA) (developed in McKinley and Cheeseman, 2017), **5.** human HeLa cell lines (human adenocarcinoma, female) separately permanently transfected and stabilized using BAC containing EG5-GFP, MKLP1-GFP, KIF4A-GFP and PRC1-GFP which were a gift from Ina Poser and Tony Hyman (Max Planck Institute of Molecular Cell Biology and Genetics, Dresden, Germany) (developed in Poser et al., 2008). Cells were grown in flasks in Dulbecco's Modified Eagle's medium (DMEM) with Ultraglutamine (1 g/l D-glucose, pyruvate) (Lonza, Basel, Switzerland) supplemented with 10% of heat-inactivated Fetal Bovine Serum (FBS) (Sigma-Aldrich, St Louis, MO, USA), and penicillin/streptomycin solution (Lonza) to a final concentration of 100 I.U./mL penicillin and 100 μg/mL streptomycin. Media was additionally supplemented for selection of some cell lines as follows: 50 μg/ml geneticin G418 (Life Technologies, Waltham, MA, USA) was added in media for various HeLa BAC cell lines described above and 500 μg/ml G418 was added in media for hTERT-RPE-1 PA-GFP-α-tubulin cell line. The induction of RPE-1 PRC1 CRISPR cell line was performed using 1 μg/mL doxycycline hyclate (Sigma) in DMEM media at 24 hr intervals for 4 days (with imaging and analysis on the fifth day), unless otherwise indicated. The cells were kept at 37°C and 5% CO_2_ in a Galaxy 170s humidified incubator (Eppendorf, Hamburg, Germany). All used cell lines were confirmed to be mycoplasma free by monthly checks using MycoAlert Mycoplasma Detection Kit (Lonza) and regular checks during imaging experiments with DNA labelling stains.

### Method details

#### Constructs, transfections and RNAi

U2OS and HeLa BAC cells were transiently transfected by electroporation using Nucleofector Kit R (Lonza, Basel, Switzerland) with the Nucleofector 2b Device (Lonza, Basel, Switzerland), using X-001 and A-028 program, respectively. Transfection protocol provided by the manufacturer was followed. Cells were transfected with mCherry-PRC1 plasmid provided by Casper C. Hoogenraad (Utrecht University, Utrecht, Netherland) or mRFP-CENP-B plasmid provided by Linda Wordeman (University of Washington, Seattle, WA, USA). 1 × 10^6^ cells were electroporated with 1.5 μg of plasmid DNA, or 100 nM PRC1 siRNA in experiments with HeLa BAC cells. Transfection of U2OS and HeLa BAC cells was performed 25–35 h before imaging. For all other siRNA treatments, 2 × 10^5^ or 3 × 10^5^ cells were seeded and cultured in 1 ml DMEM medium with same supplements (as above) at 37°C and 5% CO_2_ on 12-well cell culture plates (Greiner). After one-day growth, at ∼70% confluency cells were transfected with 200 nM (except KIF15 siRNA – 100 nM, and PRC1 siRNA – 500 nM) raw targeting or non-targeting siRNA constructs diluted in an Opti-MEM medium (Life Technologies, Waltham, MA, USA). Transfection was performed using Lipofectamine RNAiMAX Reagent (Life Technologies) using protocol provided by the manufacturer. After 5h of treatment the medium was changed to regular DMEM medium described above. After 24h of treatment cells from one well were equally reseeded into glass bottom microwells with 4 compartments (Grainer), used later for imaging. 3h prior to imaging, the medium was replaced with Leibovitz's (L-15) CO_2_-independent medium (Life Technologies), supplemented as above. The cells were imaged always 48 hours after transfection, unless otherwise indicated. The constructs used were as follows: human MKLP1 siRNA (sc-35936, Santa Cruz Biotechnology, Dallas, TX, USA), human KIF14 siRNA (sc-60882, Santa Cruz Biotechnology), human KIF4A siRNA (sc-60888, Santa Cruz Biotechnology), human KIF20A siRNA (sc-91657, Santa Cruz Biotechnology), human KIFC1 siRNA (sc-95157, Santa Cruz Biotechnology), control siRNA (sc-37007, Santa Cruz Biotechnology), human ON-TARGET KIF15 siRNA (L-004960-00-0010, Dharmacon, Lafayette, CO, USA), human ON-TARGET PRC1 siRNA (L-019491-00-0020, Dharmacon), control siRNA (D001810-10-05, Dharmacon) and human Silencer Select Validated Kif18A siRNA (s37882; Ambion, Austin, TX, USA). We observed that inhibition of MKLP1 blocked normal progression of cytokinesis that resulted in formation of binucleated cells. We have observed an increase of 81% in the number of binucleated/multinucleated cells in MKLP1 siRNA-treated samples (calculated from 88 cells) after 48h in comparison with the samples treated with control siRNA (calculated from 92 cells), similar to previous observations (Fontijn et al., 2001). Similarly, we have observed a significant increase in number of multinucleated cells following 48h treatments with MKLP2 and PRC1 siRNA (Zhu and Jiang, 2005; Neef et al., 2003). Contrary to individual depletions of MKLP1 and MKLP2, block of cytokinetic furrowing was observed in 80% of RPE-1 cells after MKLP1/MKLP2 co-depletion by 48h treatment with specific siRNAs, similar to previous report on HeLa cells (Kitagawa et al., 2013). We also observed that KIF15 siRNA-depleted cells have lower metaphase spindle length when compared to controls, as expected from the previously published data (Vanneste et al., 2009). The KIF18A depleted cells were imaged 24h post-transfection because transfection for 48h completely blocked progression of most cells to anaphase, similarly to previous reports (Stumpff et al., 2008). For inspection of the depletion levels of all target proteins by specific siRNA treatments (see Figures S2A–S2C) we performed immunocytochemistry since this method is suitable for estimation of protein depletion levels specifically on mitotic spindles using appropriate primary antibodies against same targets used in RNAi protocol and comparing localization and signal levels to non-targeting treated control cells. Cells were fixed using methanol protocol (described below) 48h after transfection with siRNA (24h for KIF18A depletion) and imaged using protocol for imaging of fixed cells (described below).

#### Drugs

The stock solution of STLC was prepared in dimethyl sulfoxide (DMSO) to a final concentration of 25 mM. Drug was obtained from Sigma-Aldrich. The working solution was prepared in DMEM at 80 μM (the half-maximal inhibitory concentration for STLC in HeLa cells is 700 nM) (DeBonis et al., 2004). At the time of treatment, the working solution was added to cells at 1:1 volume ratio to obtain a final concentration of 40 μM. To inhibit EG5, STLC was added during late metaphase (most Figures) or early anaphase (only Figures S2N, S3B, and S4A; these were single cases than were not included in the analysis of the elongation velocities). Quick response was observed as most metaphase spindles collapsed into monopolar spindles in RPE1 cells, minutes after STLC was added (Gayek and Ohi, 2014). Because of that quick collapse all experiments than included the STLC treatment were done in the “one metaphase cell per one well” regime. For immunofluorescence and expansion microscopy of alpha-Tubulin, in treatments with STLC, drug was added to the cell culture media 5 min before fixation. The stock solution of GSK-923295 was prepared in DMSO to a final concentration of 8 mM. GSK-923295 was obtained from MedChemExpress (MCE, NJ, USA). The working solution was prepared in DMEM at 200 nM. At the time of treatment, the working solution was added to cells at 1:1 volume ratio to obtain a final concentration of 100 nM (IC50 value of compound is 3.2 nM) (Wood et al., 2010). To inhibit CENP-E, GSK-923295 was added in late metaphase. Appearance of spindles blocked in prometaphase with fraction of kinetochores trapped around polar region of the spindle (Bennett et al., 2015) confirmed the effect of GSK-923295, imaged 30 min post-treatment with the drug. The stock solution of ZM 447439 was prepared in DMSO to a final concentration of 2 mM. ZM 447439 was obtained from Selleckchem (Munich, Germany). The working solution was prepared in DMEM at 8 μM. At the time of treatment, the working solution was added to cells at 1:1 volume ratio to obtain a final concentration of 4 μM (IC50 value of compound is 110 nM for Aurora A and 130 nM for Aurora B) (Ditchfield et al., 2003). To inhibit Aurora kinases, ZM 447439 was added at metaphase-to-anaphase transition. In experiment where KIF15 was inhibited by siRNA treatment in U2OS cells, we observed rapid collapse of metaphase spindle upon 40 μM STLC treatment, as reported previously (van Heesbeen et al., 2014). The stock solution of Barasertib (AZD1152-HQPA) was prepared in DMSO to a final concentration of 1 mM. The working solution for lower concentration experiment was prepared in DMEM at 120 nM and at the time of treatment, the working solution was added to cells at 1:1 volume ratio to obtain a final concentration of 60 nM (IC50 is 0.35 nM for this compound) (Yang et al., 2007). For higher concentration experiment, the working solution was prepared in DMEM at 2 μM and at the time of treatment, the working solution was added to cells at 1:1 volume ratio to obtain a final concentration of 1 μM. To inhibit Aurora B kinase, Barasertib (AZD1152-HQPA) was added at metaphase-to-anaphase transition. Appearance of abnormal prometaphase-like spindles and increased prometaphase block (Kallio et al., 2002) confirmed the effect of Barasertib (AZD1152-HQPA) on Aurora B, imaged 1 hour post-treatment with the drug. Barasertib (AZD1152-HQPA) was obtained from Selleckchem (Munich, Germany). The stock solution of Monastrol was prepared in DMSO. The working solution was prepared in DMEM at 200 μM and at the time of treatment, the working solution was added to cells at 1:1 volume ratio to obtain a final concentration of 100 μM. To inhibit EG5, monastrol was added in metaphase or early anaphase. Quick response was observed as most metaphase spindles collapsed into monopolar spindles in RPE1 cells, minutes after monastrol was added (IC50 is 50 μM for this drug) (Kapoor et al., 2000; Mayer et al., 1999). Monastrol was obtained from Sigma-Aldrich.

#### Sample preparation

When cells reached 80% confluence, DMEM medium was removed from the flask and the cells were washed with 5 mL of 1% PBS. Afterward, 1 mL of 1% Trypsin/EDTA (Biochrom AG, Berlin, Germany) was added and the cells were incubated at 37 °C and 5% CO_2_ in a humidified incubator (Eppendorf). After 5 min incubation, Trypsin was blocked by adding 2-5 mL of DMEM medium. Cells were counted using the Improved Neubauer chamber (BRAND GMBH + CO KG, Wertheim, Germany) and 4.5x10^5^ cells were seeded and cultured in 2 mL DMEM medium with same supplements (as above) at 37°C and 5% CO_2_ on 14 or 20 mm glass microwell uncoated 35mm dishes with 0.16-0.19mm (#1.5 coverglass) glass thickness (MatTek Corporation, Ashland, MA, USA). For siRNA experiments, 1x10^5^ cells were seeded in cell culture 35/10 mm glass bottom microwells with 4 compartments (Greiner, Frickenhausen, Germany) or 2x10^5^ in 12-well cell culture plates (Greiner) that were reseeded the day after into glass bottom microwells with 4 compartments. After one-day growth, 3h prior to imaging, the medium was replaced with Leibovitz's (L-15) CO_2_-independent medium (Life Technologies), supplemented with 10% FBS (Life Technologies), 100 I.U./mL penicillin and 100 μg/mL streptomycin. For live-cell staining of chromosomes 1 hour before imaging, silicon rhodamine (SiR)-DNA, also called SiR-Hoechst (Lukinavičius et al., 2015) (Spirochrome AG, Stein am Rhein, Switzerland) was added to 1 mL of cells in a DMEM medium to a final concentration of 100 nM together with efflux pump inhibitor verapamil (Spirochrome AG), only in RPE1 and U2OS cell lines, to a final concentration of 10 μM. In experiments with HeLa BAC cells 100 nM SiR-tubulin was added 1 h before imaging (Spirochrome AG). For fixation experiments, chromosomes were stained with 1 μg/mL 4′,6-diamidino-2-phenylindole (DAPI) solution.

#### Live cell imaging

HeLa cells expressing PRC1-GFP, EG5-GFP, MKLP1-GFP, and KIF4A-GFP were imaged using a Leica TCS SP8 X laser scanning confocal microscope with a HC PL APO ×63/1.4NA oil immersion objective (Leica, Wetzlar, Germany) heated with an objective integrated heater system (Okolab, Pozzuoli, NA, Italy). In primary experiments represented on most of the Figures, STLC or other drug was added on the late metaphase spindle which was then imaged with 15s intervals for 10 min or when it finished anaphase, which ever come first (the same was true for the control and siRNA-treated spindles but without the STLC addition). For excitation, a 488-nm line of a visible gas Argon laser and a visible white light laser at 580 or 652 nm were used for GFP and mRFP or SiR-tubulin, respectively. GFP and mRFP or SiR-tubulin emissions were detected with two HyD (hybrid) detectors, 498–550 nm and 580-650 or 662–692 nm, respectively. Pinhole diameter was set to 0.8 μm. Images were acquired at 2-3 focal planes with 1 μm z-spacing, 50 nm xy-pixel size, line averaging set to 3, and 400 Hz unidirectional xyzt scan mode, every 15 or 30 s. The system was controlled with the Leica Application Suite X Software (1.8.1.13759, Leica, Wetzlar, Germany) and cells were maintained at 37 °C and in 5% CO_2_ using Okolab stage top heating chamber (Okolab, Pozzuoli, NA, Italy). All RPE1 and U2OS cells were imaged using Bruker Opterra Multipoint Scanning Confocal Microscope (Buđa et al., 2017) (Bruker Nano Surfaces, Middleton, WI, USA). The system was mounted on a Nikon Ti-E inverted microscope equipped with a Nikon CFI Plan Apo VC ×100/1.4 numerical aperture oil objective (Nikon, Tokyo, Japan). During imaging, cells were maintained at 37 °C and 5% CO_2_ in Okolab Cage Incubator (Okolab). A 60 μm pinhole aperture was used and the *xy*-pixel size was 83 nm. For excitation of DAPI, GFP, mCherry or RFP and SiR fluorescence, a 405, 488, 561 and 647 nm diode laser line was used, respectively. The excitation light was separated from the emitted fluorescence by using Opterra Dichroic and Barrier Filter Set 405/488/561/640 nm (DAPI/eGFP/TRITC/Cy5) (Chroma, USA). The brightfield imaging of RPE1 PRC1 KO cells in different conditions was performed by illuminating sample with brightfield lamp (Nikon). Images were captured with an Evolve 512 Delta EMCCD Camera using 150 ms exposure time (Photometrics, Tucson, AZ, USA) with no binning performed. In experiments where whole spindle stack was imaged, z-stacks were acquired at 30-60 focal planes separated by 0.5 μm with unidirectional xyz scan mode and with “Fast Acquisition” option in software enabled. Otherwise, one or few z-stacks were imaged using 0.5 μm spacing with unidirectional xyz scan mode. The system was controlled with the Prairie View Imaging Software (Bruker Nano Surfaces).

#### Photoactivation (PA) stability and sliding assays

For photoactivation of fluorescence of PA-GFP, a 405-nm laser diode (Coherent, Santa Clara, CA, USA) was used. Photoactivation was performed using live photoactivation option in PraireView software, with duration of pulse set to 400 ms for each point and laser power set to 40% for all experiments performed. Photoactivation was performed in a line pattern on an equally distributed 10 points, where each point represents one laser hit. The interval between the points was minimal, 0.05 ms, and photoactivation area was set to 0.5 μm for each point. Photoactivation was performed after anaphase onset, perpendicular to spindle long axis, in between separating chromatids, visualized with 100 nM SiR-DNA stain (left for 30 min before imaging in the incubator) and detected using maximum power of 0.5% of 647 diode laser. SiR-DNA was used to discriminate anaphase onset by perception of peripheral sister chromatids moving into characteristic anaphase “v-shape”, which was then used as a mark for start of the photoactivation assay. PA-GFP fluorescence was detected using 488 diode laser (on 30% of a maximum laser power), turned on just after onset of anaphase to minimalize unwanted photoactivation. The excitation light was separated from the emitted fluorescence in both channels by using Opterra Dichroic and Barrier Filter Set 488/640 nm (eGFP/Cy5) (Chroma, USA). The interval between consecutive frames was set to 0.8 s, imaging one central z-plane and program was set to record 200 consecutive frames.

#### Washout of STLC

Human RPE1 cell line stably expressing centrin1-GFP and CENP-A-GFP were grown on glass-bottomed dishes (14 mm, No. 1.5, MatTek Corporation) and transfected with 200 nM KIF4A siRNA as described in the Section “Constructs, transfections and RNAi”. After 48 hours cells were ready for live cell imaging. To inhibit EG5, 80 μM STLC diluted in DMEM was added to dish during imaging of metaphase or early anaphase cells and imaging started immediately. The working solution of 80 μM STLC was added to cells at 1:1 volume ratio to obtain a final concentration of 40 μM. After 120-300 seconds, imaging was stopped and cell media with STLC was removed with vacuum aspirator pump. After that, new, 5 mL of the warm cell media without STLC was added to the cells and imaging was continued.

#### Immunofluorescence

Human RPE-1 cell line stably expressing centrin1-GFP and CENP-A-GFP were grown on glass-bottomed dishes (14 mm, No. 1.5, MatTek Corporation) and fixed by 1 mL of ice-cold methanol for 3 min at -20°C for visualization of PRC1, EG5, KIF15, KIF4A, MKLP1, MKLP2 and KIF14. To visualize alpha-tubulin in control cells and in the treatments, ice-cold methanol protocol was avoided because it destroyed unstable fraction of microtubules and cells were instead fixed by a microtubule-preserving mixture of 3.2% PFA (paraformaldehyde) and 0.25% GA (glutaraldehyde) in microtubule-stabilizing PEM buffer (0.1 M PIPES, 0.001 M MgCl_2_ x 6 H_2_O, 0.001 M EDTA, 0.5 % Triton-X-100) for 10 min at room temperature (Heuser and Kirschner, 1980). After fixation with PFA and GA, for quenching, cells were incubated in 1mL of freshly prepared 0.1% borohydride in PBS (phosphate-buffered saline) for 7 min and after that in 1 mL of 100 mM NH_4_Cl and 100 mM glycine in PBS for 10 min at room temperature. Both methanol fixed cells and PFA and GA fixed cells were then washed with 1 mL of PBS, 3 times for 5 min. To block unspecific binding of antibodies, cells were incubated in 500 μL blocking/permeabilization buffer (2% normal goat serum (NGS) and 0.5% Triton-X-100 in water) for 45 min at room temperature. Cells were then incubated in 500 μL of primary antibody solution for 24h at 4 °C. The following primary antibodies were used: mouse monoclonal PRC1 (sc-376983, Santa Cruz Biotechnology), diluted 1:50; mouse monoclonal EG5 (sc-365681, Santa Cruz Biotechnology), diluted 1:50; mouse monoclonal KIF4A (E-8, sc-365144, Santa Cruz Biotechnology), diluted 1:50; rabbit polyclonal MKLP-1 (sc-867, Santa Cruz Biotechnology), diluted 1:50; rabbit polyclonal KIF20A (A300-879A, Bethyl Laboratories, TX, USA), diluted 1:500; rat anti-alpha Tubulin YL1/2 (MA1-80017, Invitrogen, CA, SAD), diluted 1:500; rabbit polyclonal KIF14 (ab3746, Abcam), diluted 1:50; mouse monoclonal KIFC1 (M-63, sc-100947, Santa Cruz Biotechnology), diluted 1:50; rabbit polyclonal KIF18A (A301-080A, Bethyl Laboratories), diluted 1:100 and rabbit polyclonal KIF4A (A301-074A, Bethyl Laboratories), diluted 1:100. After primary antibody, cells were washed in PBS and then incubated in 500 μL of secondary antibody solution for 45 min at room temperature. Following secondary antibodies were used: donkey anti-mouse IgG Alexa Fluor 594 (ab150112, Abcam, Cambridge, UK), donkey anti-rabbit IgG Alexa Fluor 594 (ab150064, Abcam), donkey anti-rabbit IgG Alexa Fluor 647 (ab150075, Abcam), donkey anti-mouse IgG Alexa Fluor 647 (ab150107, Abcam) and donkey anti-rat IgG Alexa Fluor 594 (ab150156, Abcam), all diluted 1:1000. Finally, cells were washed with 1 mL of PBS, 3 times for 5 min.

#### Expansion microscopy

Expansion microscopy protocol was a custom made (Ponjavić et al., 2021) as a combination of optimized steps from of various previously developed protocols for expansion microscopy of human cells in culture (Truckenbrodt et al., 2019; Chozinski et al., 2016; Tillberg et al., 2016). Human RPE-1 cell line stably expressing centrin1-GFP and CENP-A-GFP were grown on glass-bottomed dishes (14 mm, No. 1.5, MatTek Corporation) and fixed by a mixture of 3.2% PFA (paraformaldehyde) and 0.25% GA (glutaraldehyde) in PEM buffer (0.1 M PIPES, 0.001 M MgCl_2_ x 6 H_2_O, 0.001 M EDTA, 0.5% Triton-X-100) for 10 min at room temperature. After fixation with PFA and GA, for quenching, cells were incubated in 1mL of freshly prepared 0.1% borohydride in PBS (phosphate-buffered saline) for 7 min and after that in 1 mL of 100 mM NH_4_Cl and 100 mM glycine in PBS for 10 min at room temperature. Cells were then washed with 1 mL of PBS, 3 times for 5 min. To block unspecific binding of antibodies, cells were incubated in 500 μL blocking/permeabilization buffer (2% normal goat serum (NGS) and 0.5% Triton-X-100 in water) for 45 min at room temperature. Cells were then incubated in 500 μL of primary antibody solution for 24h at 4 °C. Primary antibody that was used is rat anti-alpha Tubulin (MA1-80017, Invitrogen). After primary antibody, cells were washed in PBS and then incubated in 500 μL of secondary antibody solution for 45 min at room temperature. Secondary antibody used was donkey anti-rat IgG Alexa Fluor 594 (1:1000, ab150156, Abcam). To remove unbound secondary antibody from the sample, cells were washed three times with 1 ml of PBS for 5 min each, at room temperature. Acryloyl-X 1:100 (vol/vol) (Thermo Fisher Scientific) was diluted to 0.1 mg/ml in anchoring buffer. Sample was incubated with the anchoring solution for at least 6 h or overnight. Sample was washed two times in 1 mL of PBS, 5 min each, at room temperature, right before application of the gelation solution. The gel monomer components were mixed together (8.6 wt% Sodium acrylate, 2.5 wt% Acrylamide, 0.15 wt% N,N’-Methylenebisacrylamide, 11.7 wt% Sodium chloride, 1x PBS and water) and the polymerization solution was applied to the sample. The gel was left to polymerize by incubation for 1 h in a humidified chamber at 37 °C. After, the sample was incubated in the digestion buffer (50 mM Tris pH 8.0, 1 mM EDTA, 0.5% Triton-X-100, 0.8 M guanidine HCl) with proteinase K added last (1:100, final concentration 8 units/mL) for >8 h, or overnight in a humidified digestion chamber, at room temperature. After digestion, the remaining digestion buffer was removed from the dish and at least 2 mL of ddH_2_O was added. ddH_2_O was removed after 10–20 min of incubation, using a Pipetboy or a vacuum pump. Fresh ddH_2_O was added and these steps were repeated until no further expansion of the gel can be observed (usually after four to five water exchanges). Before the imaging, excess water was removed by pipette and after that by placing filter paper in the corners of the dish to minimalize movement of the gel during imaging. Expansion factor was estimated from measured spindle length of the expanded sample after dividing it with the spindle length of the non-expanded spindle in the same phase of the anaphase. Expansion factor was estimated from the spindle lengths to be around 2.3x, calculated as a ration between anaphase spindle lengths before and after expansion.

#### Western blotting

For western blotting, untreated or cells following treatments were collected from a 25cm^2^ flask and lysed in RIPA buffer (R0287, Sigma) containing 1x protease inhibitor (5892970001, Roche), 1x phosphatase inhibitor (4906837001, Roche) and 1mM PMSF by two cycles of freezing and thawing in liquid nitrogen. Protein extracts collected after centrifugation were subjected to SDS-PAGE and transferred onto nitrocellulose membrane (IB23002, Invitrogen). After blocking with PBS containing 0.1% Tween-20 and 5% milk, membranes were incubated overnight with primary antibodies. Immunoblots were performed using the following primary antibodies: mouse anti-KIF4A (1:1000, Santa Cruz, sc-365144), mouse anti-PRC1 (1:500, Santa Cruz, sc-376983), mouse anti-MKLP1 (1:1000; Santa Cruz, sc-390113), mouse anti-KIF20A (D-3, sc-374508, Santa Cruz) mouse anti-GAPDH (diluted 1:1000, Abcam, ab9485) and mouse anti-beta-actin (1:1000; Invitrogen, PA1-183). Membranes were then washed in PBS containing 0.1% Tween-20 and incubated 1 h with HRP-conjugated goat anti-mouse secondary antibodies (dilution 1:10000, ab205719, Abcam). After washing with PBS containing 0.1% Tween-20, immunodetection was performed using C-DiGit blot scanner (LI-COR, Bad Homburg, Germany) with WesternSure PREMIUM Chemiluminescent Substrate (926-95000, LI-COR). Western blots of KIF20A siRNA treated cells (using both Santa Cruz sc-91657 and Dharmacon ON-TARGETplus SMARTpool L-004957 siRNAs) yielded very low levels of protein depletion when compared to the control cells, contrary to the results obtained by the immunofluorescence using Santa Cruz sc-91657 siRNA and anti-MKLP2 antibody (A300-879A, Bethyl Laboratories) (Figure S2).

#### Imaging of fixed cells

All RPE-1 and U2OS fixed cells were imaged using Bruker Opterra Multipoint Scanning Confocal Microscope (Bruker) described above. In experiments where whole spindle stack was imaged, z-stacks were acquired at 30-60 focal planes for immunofluorescence images, and 60-120 focal planes for expanded samples, separated by 0.5 μm with unidirectional xyz scan mode. A 60 μm pinhole aperture was used and the *xy*-pixel size was 83 nm. For excitation of DAPI, GFP, mCherry or RFP and SiR fluorescence, a 405, 488, 561 and 647 nm diode laser line was used, respectively. The excitation light was separated from the emitted fluorescence by using Opterra Dichroic and Barrier Filter Set 405/488/561/640 nm (DAPI/eGFP/TRITC/Cy5) (Chroma). Images were captured with an Evolve 512 Delta EMCCD Camera using 300ms exposure time (Photometrics, Tucson, AZ, USA) with no binning performed. The frame average was performed 8 times for immunofluorescence images and 16 times for expansion microscopy images. All experiments were carried out using Nikon CFI Plan Apo VC ×100/1.4NA oil objective (Nikon).

#### STED microscopy

STED images of U2OS and RPE-1 cells were recorded at the Core Facility Bioimaging at the Biomedical Center, LMU Munich. STED resolution images were taken of SiR-tubulin signal, whereas GFP signal of kinetochores was taken at confocal resolution. Gated STED images were acquired with a Leica TCS SP8 STED 3X microscope with pulsed white light laser excitation at 652 nm and pulsed depletion with a 775 nm laser (Leica, Wetzlar, Germany). The objective used was HC PL APO CS2 ×93/1.30NA GLYC with a motorized correction collar set to 63%. Scanning was done at 30 Hz, a pinhole setting of 0.93 AU (at 580 nm), and the pixel size was set to 33.29 × 33.29 nm. The signals were detected with Hybrid detectors with the following spectral settings: SiR-tubulin (excitation 652; emission: 662–692 nm; counting mode, gating: 0.35–6 ns) and GFP (excitation 488; emission 498–550; counting mode, gating: 0.50-6 ns). STED 775 nm laser was delayed by -150 ps. Cells were stained with SiR-tubulin dye at 100 nM concentration, 1 h before imaging.

#### EB3 imaging

Live imaging of RPE-1 cells stably expressing EB3-GFP and H2B-mCherry was performed on a spinning disk confocal microscope system (Dragonfly, Andor Technology, Belfast, UK) using 63x/1.47NA HC PL APO oil objective (Leica) and Zyla 4.2P scientific complementary metal oxide semiconductor (sCMOS) camera (Andor Technologies). During imaging cells were maintained at 37°C and 5% CO2 within H301-T heating chamber (Okolab). Images were acquired using Fusion software (v 2.2.0.38). For excitation, 488-nm and 561-nm laser lines were used for visualization of GFP, and mCherry, respectively. Only one spindle central z plane (plane where both centrosomes could be discerned) was acquired sequentially with both laser lines, every 1 s with 150 ms exposure time for 10 min. Control, KIF4A siRNA depleted and KIF4A siRNA depleted STLC treated cells were imaged with the same imaging protocol.

### Quantification and statistical analysis

No statistical methods were used to predetermine sample size. The experiments were not randomized and, except where stated, investigators were not blinded to allocation during experiments and outcome assessment.

#### Parameters used to define metaphase-to-anaphase transition

The time of anaphase A onset for each individual cell was defined as the time point immediately prior to the separation of sister chromatid populations as annotated manually using visual inspection based on the increased distance between the sister kinetochore groups as cells transitions from metaphase to anaphase. The onset of spindle elongation was defined as the time point immediately prior to the separation of two centrosomes as annotated by checking for continuous increase in the spindle length for two consecutive frames using custom made Matlab script (The MathWorks Inc., USA, R2018a) and double checked by visual inspection of original movies for possible errors.

#### Tracking and quantification of kinetochore, centrosome and chromosome motions

Kinetochores and centrosomes were tracked in time using Low Light Tracking Tool (LLTT), an ImageJ plugin (Krull et al., 2014). Tracking of kinetochores in the x, y plane was performed on individual imaging planes or on maximum-intensity projections of up to three planes. The position in z direction (3D) was ignored because it had a small contribution to the kinetochore movement. In order to obtain optimal tracking results, it was necessary to define good intensity offset in the channel with fluorescently labelled kinetochores. The intensity offset was defined by measuring the mean intensity around kinetochores in the first frame before start of tracking using ‘freehand selection’ tool in Fiji. Sometimes, when photobleaching was prominent, bleach correction using Histogram Matching Method in Fiji was done to compensate for a decrease in background intensity in time. Also, it was necessary to define EMCCD gain and Electrons per A/D count of the used EMCCD camera to correct the measured flux of the object and background noise. The EMCCD-GaussianML tracking algorithm method was used (Krull et al., 2014) because it yielded more precise results compared with Gaussian-ML method, especially in situations when fast movement of the tracked object occurred (on a scale of micron per frame or more). All tracked objects were double checked by eye to ensure that tracking was accurate, because it was inaccurate in situations of an uneven intensity of tracked objects and in situations when multiple similar objects appeared in close proximity. If those cases were predominant, tracking was performed manually extracting xyz-coordinates of each kinetochore. σ value (standard deviation of the Gaussian used to approximate the Point Spread Function (PSF) of the tracked objects) was set to 1 to encompass just the tracked kinetochore. Detailed quantitative analysis of centrosome and kinetochore location was performed using custom made MATLAB scripts which determines the distance between the centroid of the sister kinetochores. Chromosomes were tracked using ImageJ line tool from a centromere region of a one sister chromosome to the same region of another sister. The velocities of kinetochore and centrosome separation were measured between 1 and 3 minutes after the onset of anaphase, (d_3_-d_1_)/(2t) where d is the distance between sister kinetochore centroids subtracted to the same distance in the last frame before anaphase or pole-to pole distance subtracted to the same distance in the last frame before anaphase (termed Δ for all parameters defined in this manner) and t is the time period. This interval was chosen because measured parameters are characterized by linear increase during this time period in untreated human cells (Su et al., 2016; Afonso et al., 2014), justifying the usage of linear fitting to obtain the velocities. The raw parameters (sister kinetochore, centrosome or poleward kinetochore-to-centrosome distance) were plotted as a function of time in Matlab home written script and on each curve linear regression was performed for points starting from 60s after anaphase onset, to the 120s after. The velocities of chromosome motion, in photoactivation experiments, were calculated from the onset of photoactivation in anaphase (determined by the SiR-DNA staining) to the 60s after. In CRISPR experiments, velocities of chromosome motion were calculated within first minute of anaphase. The velocities were calculated from a slope of a regression line. Position of the sister chromatid in every time-frame was determined at the leading edge of the moving chromatid where kinetochore is located.

#### Quantification of microtubule stability

Microtubule stability was measured on single z-plains acquired in photoactivation assay. The onset of photoactivation was set as a first point of measurement, after all dots finished on predetermined photoactivation line, and second point of measurement was placed in a frame 30s after onset in all conditions imaged. Region between separated chromosomes in the last frame was used as a borderline for drawing a 100px thick line in ImageJ, horizontal to the spindle long axis, from which the fluorescence profile was extracted. The mean background fluorescent intensity measured in the same frame in cytoplasm (measured in ImageJ by drawing a 100px thick line), was subtracted from obtained intensities. The obtained values were plotted in SciDavis program (Free Software Foundation, Inc., Boston, MA, USA) and area under the peak was linearly integrated to obtain area of intensity for that frame. The same measurement was performed for the frame defined as the onset of photoactivation using region of the same dimensions. The two values were divided to give estimation on how much of photoactivated-α-tubulin fluorescence was lost following 30 s period in all conditions tested giving estimation of relative microtubule turn-over and stability.

#### Quantification of sliding velocity

The hTERT-RPE-1 permanently transfected and stabilized using PA-GFP-α-tubulin where imaged in a one middle z-plane using unidirectional xyz scan mode and with “Fast Acquisition” option in software enabled with interval of 0.8s between two frames. The width of photoactivated spot in the spindle midzone was measured using ImageJ by drawing a line segment along the photoactivated region, from the moment when photoactivation of tubulin finished for all dots on line segment used for photoactivation, across time frames separated by 0.8s, to the last frame of measurable photoactivation signal in the spindle midzone. The brightness and contrast were adjusted in every frame on original 16-bit images to clearly see the ends of bright photoactivated signal within the midzone. The width was plotted as a function of time in Matlab home written script and on each curve linear regression was performed for points starting from 0, representing start of a measurement, to the 30s after. The velocities where calculated from a slope of a regression line. The position of a centrosome was determined only in those cells in which signal of photoactivated α-tubulin could be discerned on both poles. Multiple bundles were tracked in single cell where appropriate but their velocities values were averaged for correlation graphs and bar plots.

#### Quantification of PRC1 overexpression

The fluorescence intensity signal of PRC1-mCherry was measured on the whole spindle region using ImageJ polygon selection on sum-intensity projection of all z-stacks acquired. The background fluorescence intensity measured in the cytoplasm was subtracted from the mean value obtained, and this value was divided with number of z-stacks used in sum projection.

#### Quantification of integrated signal intensity of midzone proteins

The fluorescence intensity signal of midzone proteins was measured in the midzone region of the mid- and late-anaphase spindles ranging between 17-27 μm in length using ImageJ “Rectangle selection” tool (width 40 px, length 10 μm) on sum-intensity projection of all acquired z-planes. The line was set perpendicularly to long spindle axis and was placed in the middle between the two sets of separating chromosomes. The background fluorescence intensity measured in the cytoplasm was subtracted from the obtained values, and calculated integrated intensity value was divided with number of z-stacks used to generate the sum projection of each cell. The obtained integrated intensity values for each group was normalized to the mean value of control non-targeting siRNA-treated group for each target protein.

#### Quantification of integrated tubulin midzone intensity signal

The fluorescence intensity signal of α-tubulin from immunofluorescent images was measured in the midzone region of the mid- and late anaphase spindles using ImageJ “Straight line” tool (width 40 px, length 10 μm) on sum-intensity projections of all acquired z-planes. The line was set perpendicularly to long spindle axis and was placed in the middle between the two sets of separating chromosomes. The background fluorescence intensity was defined as the minimum value obtained on the line across the midzone and this value was subtracted from the obtained initial values along the line. Calculated value of integrated intensity was divided with number of z-stacks used to make the sum projection for each cell. The obtained integrated intensity was normalized to the mean value of the control group for each treatment.

#### Quantification of the length of astral microtubules

The 3D length of astral microtubules was measured on the single z-planes of tubulin-labeled anaphase spindles ranging between 12-18 μm in length, as assessed by measuring length between centrosomes, using “Multi-point” tool in ImageJ to track the microtubule point-by-point (points separated by ∽1 μm). Only those astral microtubules were measured that contacted the cell membrane boundary with their tip and that were projected opposite of the other spindle pole.

#### Quantification of EB3 comet parameters

EB3 comets were discerned using TrackMate ImageJ tracking plugin. Images where first rotated in a way long spindle axis was set in a perpendicular view and astral and midzone regions where defined using ImageJ “Segmented Line” tool. Each astral region was defined as a region enclosed by a line passing through the center of each centrosome perpendicular to long spindle axis and by a continuous line following the cortical cell membrane boundary (Figure S). Midzone region was defined in each frame as a rectangular region defined by the edges of the H2B chromosome signal and the edge of the spindle EB3 signal. The EB3 comets were recognized using LOG (Laplacian of Gaussian) detector with the “Estimated blob diameter” of 1 μm and “Threshold” value of 0.8 μm (with sub-pixel localization enabled). The presented parameters were optimized in a way that no more than 5% of recognized EB3 comets were present outside the cell boundaries in each time-frame. The velocity and stability of EB3 comets was tracked from the whole-spindle kymographs generated using 40-pixel thick “Straight line” in ImageJ and by applying “KymographBuilder” ImageJ plugin. The length and stability were measured on discernable astral and midzone comets by using ”Polygon” selection tool in ImageJ where EB3 comet track is hypothenuse of the right-angled triangle defined by the “Polygon” tool.

#### CRISPR KO cell scoring

CRISPR PRC1 KO cell were scored for successful knockout of PRC1 5 days post-induction with doxycycline by fixing cells with cold-methanol protocol (described above) and staining with mouse monoclonal PRC1 antibody (sc-376983, Santa Cruz Biotechnology), diluted 1:50, and Alexa Fluor 488 secondary antibody (diluted 1:1000) (Invitrogen). SiR-DNA, 100nM final concentration, was added post-fixation, 30 min before imaging, to stain DNA in order to identify anaphase cells. Successful KO without noticeable PRC1 signal in the spindle midzone was observed in 90% of analyzed cells (60 out of 68 cells) and 10% of the cells imaged had a normal PRC1 appearance, localizing to the spindle midzone, similarly to all non-induced controls imaged.

#### Image processing and statistical analysis

Image processing was performed in ImageJ (National Institutes of Health, Bethesda, MD, USA). Quantification and statistical analysis were performed in MatLab. Figures were assembled in Adobe Illustrator CS6 and Adobe Photoshop CS6 (Adobe Systems, Mountain View, CA, USA). All kymographs (except for those on Figure 6) were generated in ImageJ on maximum intensity projections of vertically rotated images by using a “Reslice” command and them performing maximum intensity projection and montage images were generated in ImageJ in single focal plane vertically oriented images containing only one photoactivated bundle using “Make Montage” command. The kymograph and montage images were rotated in every frame to fit the long axis of the spindle to be parallel with the central long axis of the box in ImageJ and spindle short axis to be parallel with the central short axis of the designated box in ImageJ. The designated box sizes were cut in the same dimensions for all panels in Figures where the same experimental setups were used across the treatments. When comparing different treatments in channels with same proteins labelled, minimum and maximum of the intensity in that channel was set to the values in the control treatment. When indicated, smoothing of images was done using "Gaussian blur" function in ImageJ (s=1.0-1.5). Color coded maximum intensity projections of the z-stacks were done using "Temporal color code" tool in Fiji by applying 16 color lookup-table (LUT) or other LUT as indicated. For generation of univariate scatter plots, the open "UnivarScatter" Matlab extension was used (https://github.com/manulera/UnivarScatter). Data are given as mean ± standard deviation (s.t.d.), unless otherwise stated. Other dispersion measures used are defined in their respective figure captions or on the Figure 1 and Figure S1 if the same measures are used across all figures. The exact values of n are given in respective figure captions, where n represents the number of cells or the number of kinetochore pairs tracked, as defined for each n in figure captions or tables. The number of independent experiments is also given in figure captions. p values were obtained using unpaired two-sample Student’s t-test (significance level was 5%). When comparing the same parameters cell by cell, we used paired two-sample Student’s t-test (significance level was 5%). p < 0.05 was considered statistically significant, very significant if 0.001 < p < 0.01 and extremely significant if p < 0.001. Values of all significant differences are given with degree of significance indicated (^∗^0.01 <p < 0.05, ^∗∗^0.001 < p < 0.01, ^∗∗∗^p < 0.001, ^∗∗∗∗^ < 0.0001). For linear regression correlation measure between two parameters, nonparametric Spearman correlation coefficient, termed rs, was used where p<0.001, calculated using “corr” function in Matlab (Statistics Toolbox R14). The number of analyzed cells and specific parameters are given in the respective figure panels and Figure S1A.

## Supplemental information

Document S1. Figures S1–S7

Document S2. Article plus supplemental information
