# Supplementary material for: Microtubule-sliding modules based on kinesins EG5 and PRC1-dependent KIF4A drive human spindle elongation
Source: Dev Cell. 2021 May 3;56(9):1253–1267.e10. doi: 10.1016/j.devcel.2021.04.005 (PMC8098747; doi:10.1016/j.devcel.2021.04.005)
Supplement: Document S1. Figures S1–S7 [file mmc1.pdf]

Developmental Cell, Volume *56*

## Supplemental information

# Microtubule-sliding modules based on kinesins EG5 and PRC1-dependent KIF4A drive human spindle elongation

**Kruno Vukušić, Ivana Ponjavić, Renata Buđa, Patrik Risteski, and Iva M. Tolić**

**A**

| Condition | CEN-CEN | n | p |
|---|---|---|---|
| Control | 2.36 ± 0.08 | 33 | n.a. |
| EG5 inh. | 2.07 ± 0.18 | 16 | n.s. |
| KIF15 | 1.69 ± 0.27 | 10 | $2.80x10^{-3}$ |
| KIF15+EG5 inh. | 1.13 ± 0.26 | 9 | $1.56x10^{-6}$ |
| PRC1 | 2.41 ± 0.22 | 17 | n.s. |
| PRC1+EG5 inh. | 0.55 ± 0.19 | 10 | $2.36x10^{-12}$ |
| KIF4 | 2.08 ± 0.17 | 10 | n.s. |
| KIF4+EG5 inh. (STLC) | 0.35 ± 0.17 | 15 | $7.26x10^{-16}$ |
| KIF4+EG5 inh. (MONASTROL) | 0.37 ± 0.18 | 8 | $4.62x10^{-13}$ |
| MKLP1 | 1.67 ± 0.11 | 15 | $1.78x10^{-5}$ |
| MKLP1+EG5 inh. | 1.50 ± 0.18 | 10 | $2.44x10^{-5}$ |
| MKLP2 | 1.30 ± 0.20 | 6 | $1.59x10^{-5}$ |
| MKLP2+EG5 inh. | 1.60 ± 0.18 | 12 | $1.01x10^{-4}$ |
| MKLP1+MKLP2 | 1.14 ± 0.07 | 17 | $8.25x10^{-13}$ |
| MKLP1+MKLP2+EG5 inh. | 0.89 ± 0.25 | 12 | $8.6x10^{-10}$ |
| MKLP1+MKLP2+KIF4 | 1.68 ± 0.13 | 11 | $1.37x10^{-4}$ |
| CENP-E | 1.52 ± 0.18 | 8 | $7.63x10^{-5}$ |
| CENP-E+EG5 inh. | 2.03 ± 0.24 | 7 | n.s. |
| KIF14 | 1.45 ± 0.08 | 7 | $1.71x10^{-5}$ |
| KIF14+EG5 inh. | 1.70 ± 0.14 | 6 | $2.6x10^{-3}$ |
| HSET | 2.01 ± 0.19 | 11 | n.s. |
| HSET+EG5 inh. | 2.35 ± 0.19 | 11 | n.s. |
| KIF18A | 1.96 ± 0.20 | 7 | n.s. |
| KIF18A+EG5 inh. | 1.05 ± 0.19 | 10 | $1.43x10^{-8}$ |
| AURB(L) | 1.42 ± 0.19 | 7 | $7.35x10^{-4}$ |
| AURB(L)+EG5 inh. | 1.28 ± 0.13 | 6 | $1.51x10^{-7}$ |
| AURB(H) | 1.10 ± 0.17 | 7 | $5.70x10^{-6}$ |
| AURB(H)+EG5 inh. | 1.09 ± 0.34 | 9 | $2.97x10^{-6}$ |
| AURA,B+EG5inh. | 0.86 ± 0.13 | 8 | $2.04x10^{-9}$ |

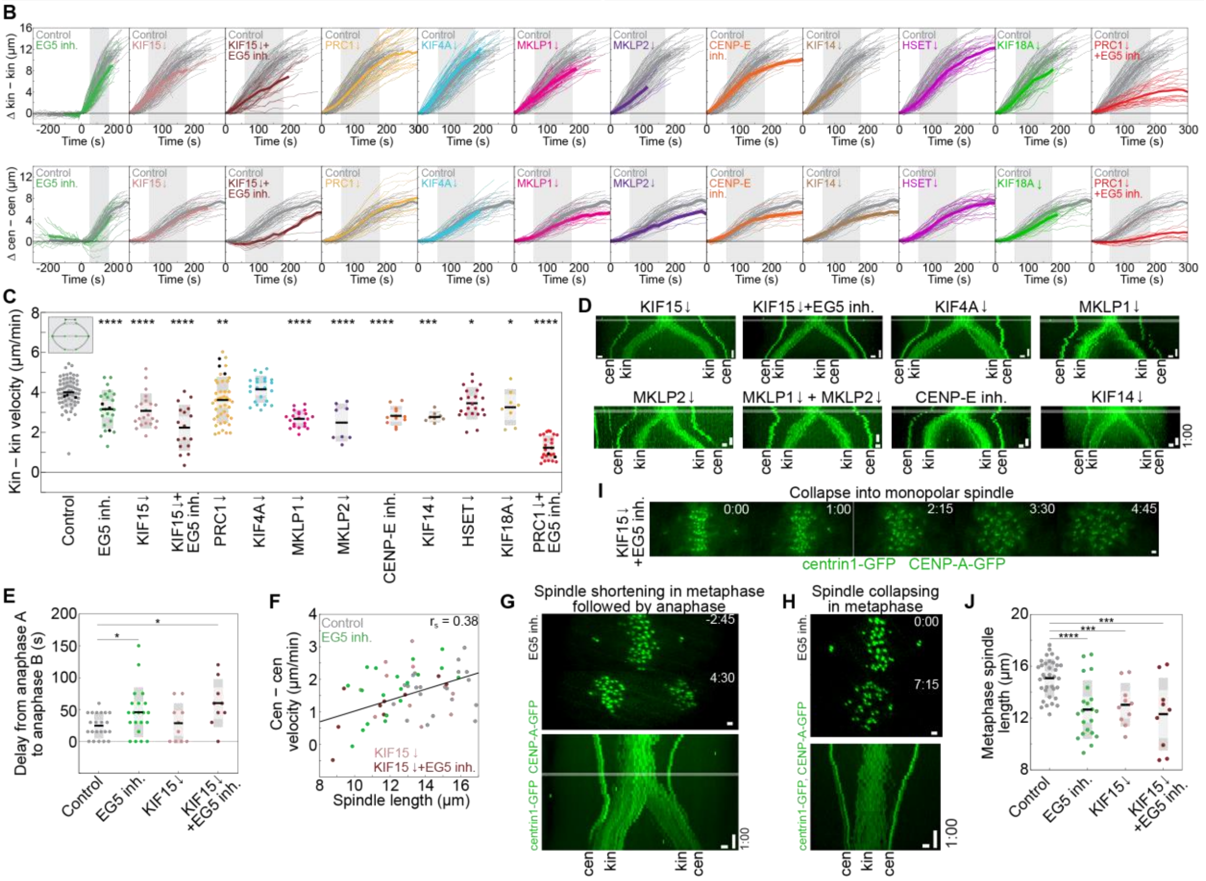


**Figure S1. Perturbation of PRC1 when combined with EG5 inhibition blocks anaphase spindle elongation, whereas individual perturbations of EG5-KIF15 proteins do not, Related to Figure 1.** (**A**) Table of spindle elongation velocities (cen-cen, in μm/min) for all conditions presented in the article with the number of measured cells and p-values when compared with the control group using Student t-test. n is equal to the number of cells. (**B**) Plots of relative kinetochore and centrosome separation distance (Δ) defined as the kinetochore-to-kinetochore (kin-kin, top panel) and centrosome-to-centrosome (cen-cen, bottom panel) distance at time t minus the kin-kin and cen-cen distance at t = 0, over time for indicated treatments. Individual kinetochore and centrosome pairs (thin lines), mean (thick lines). Grey shaded regions represent the time frame (60-180s from the anaphase onset) in which velocity measurements were performed. Number of kinetochore pairs: 89 in control, 55 in EG5-inhibited, 28 in KIF15-depleted, 24 in KIF15-depleted EG5-inhibited, 51 in PRC1-depleted, 37 in KIF4A-depleted, 43 in MKLP1-depleted, 8 in MKLP2-depleted, 18 in CENP-E-inhibited, 8 in KIF14-depleted, 22 in HSET-depleted, 8 in KIF18A depleted and 25 in PRC1-depleted EG5-inhibited cells. Number of cells: 33 in control, 21 in EG5-inhibited, 10 in KIF15-depleted, 9 in KIF15-depleted EG5-inhibited, 17 in PRC1-depleted, 11 in KIF4A-depleted, 16 in MKLP1-depleted, 6 in MKLP2-depleted, 7 in CENP-E-inhibited, 8 in KIF14-depleted, 11

in HSET-depleted, 8 in KIF18A-depleted and 10 in PRC1-depleted EG5-inhibited cells. At least three independent experiments for every condition regarding siRNAs or non-targeting treatments except KIF14 siRNA and MKLP2 siRNA which were done in two independent experiments, while number of independent experiments regarding STLC and GSK-923295 treatment is equal to the number of cells. Time zero represents anaphase onset. (**C**) Quantification of the velocity of separation of sister kinetochores (kin-kin, see scheme) in the indicated treatments. Boxes represent the standard deviation (dark grey), 95% confidence interval of the mean (light grey) and mean value (black) for each condition. Statistics: t-test (*P < 0.05, **P < 0.01, ***P < 0.001, ***P < 0.0001). Number of kinetochore pairs and cells for each treatment is the same as in (A). (**D**) Kymographs made from live images of RPE-1 cells from different perturbations as indicated. Horizontal grey lines in the kymographs indicate the onset of anaphase. (**E**) Quantification of spindle elongation (anaphase B) delay calculated as the difference between spindle elongation onset and kinetochore separation onset (anaphase A). Note: EG5 inhibition by STLC treatment, with or without depletion of KIF15, induces a small delay in spindle elongation start compared to controls. Numbers: 22 control, 21 STLC-treated, 10 KIF15-depleted and 9 KIF15-depleted STLC treated cells. (**F**) Distribution of spindle elongation velocity (cen-cen) versus initial spindle length, measured at the onset of anaphase, in the indicated conditions and linear regression (line); $r_s$, Spearman correlation coefficient, P < 0.01. Numbers: same as in (E). (**G**) Live images (top) and corresponding kymograph (bottom) of STLC-treated RPE-1 cell stably expressing CENP-A-GFP and centrin1-GFP show shortening of the spindle in metaphase and normal spindle elongation in anaphase. Horizontal grey line in the kymograph indicates the onset of anaphase. (**H)** Live images (top) and corresponding kymograph (bottom) of RPE-1 cell show the spindle collapsing during metaphase after STLC treatment. Time zero represents the start of STLC treatment. Images are the maximum projection of the acquired z-stack. kin-kinetochore and cen-centrosome. (**I)** Live images of KIF15 siRNA depleted STLC-treated RPE-1 cell show metaphase spindle collapse and monopolar spindle formation. Time zero represents start of STLC treatment. (**J)** Quantification of peak metaphase spindle length in various conditions as indicated (n=35 cells in control, 21 in STLC-treated, 10 in KIF15 siRNA-depleted and 9 in KIF15 siRNA-depleted STLC-treated). Note: KIF15 siRNA-depleted cells have lower metaphase spindle length when compared to controls, as expected from previously published data (Vanneste et al., 2009). Time is shown as minutes:seconds. Depletion is indicated by an arrow pointing down. Horizontal scale bars, 1 µm. Vertical scale bars, 1min.

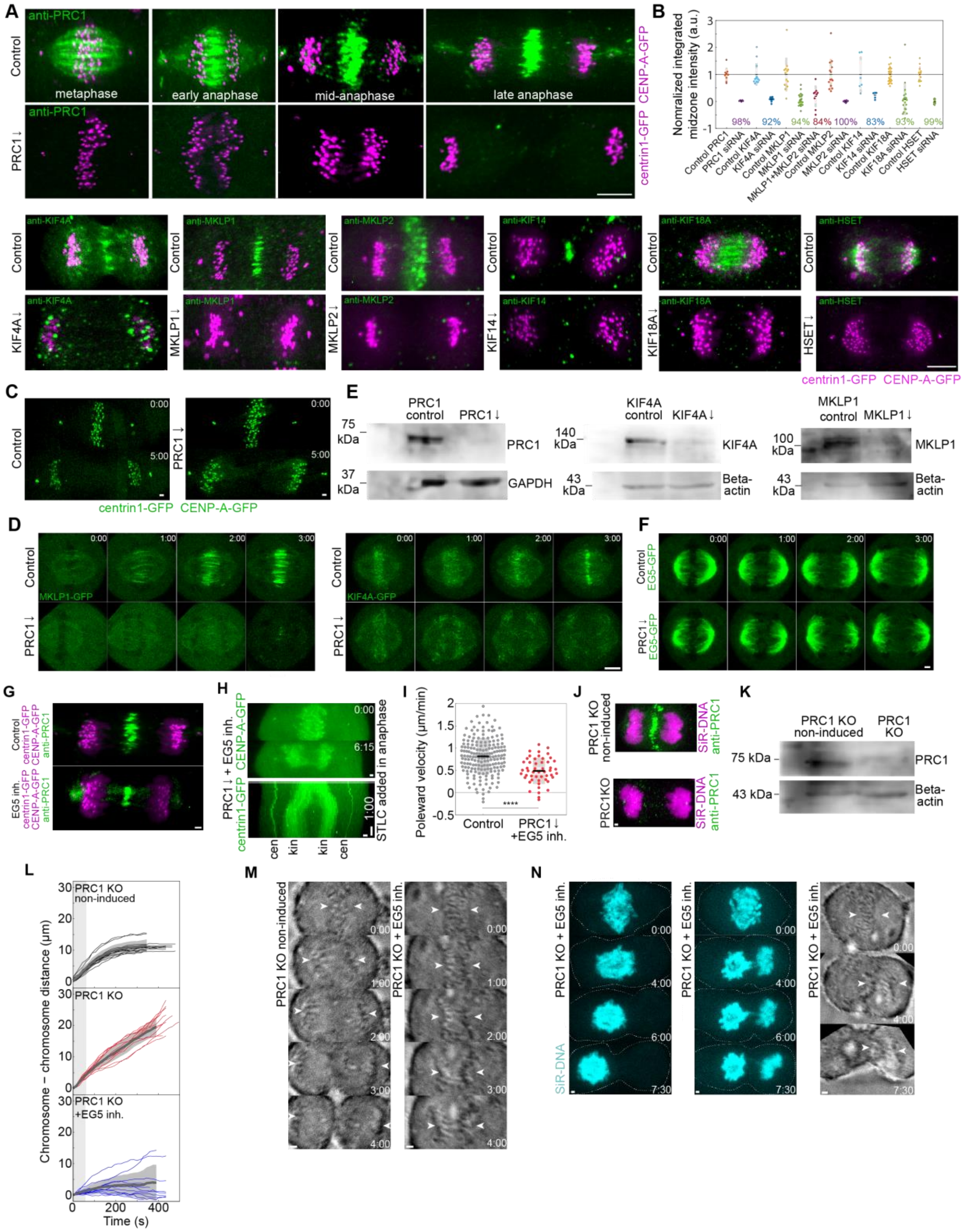


**Figure S2. PRC1 and EG5 are independent from each other during anaphase and PRC1 CRISPR knock-out (KO) combined with EG5 inhibition leads to unsuccessful chromosome segregation in anaphase, Related to Figures 1 and 2.** (**A**) Immunofluorescence images of fixed non-targeting treated and PRC1 siRNA-depleted RPE-1 cells stably expressing CENP-A-GFP and centrin1-GFP (magenta) stained with AlexaFluor594 conjugated with PRC1 antibody (green). Note: PRC1 was efficiently depleted by siRNA-treatment in all phases of mitosis as depicted (top). Immunofluorescence images of fixed RPE-1 cells stably expressing CENP-A-GFP and centrin1-GFP (magenta) and stained with AlexaFluor594

conjugated with an antibody specific for the indicated target protein (green) in non-targeting treatments (top row) and in treatment with indicated siRNAs (bottom row) (bottom). Scale bars, 5 µm. (**B**) Quantification of the normalized midzone intensity of target proteins from immunofluorescent images in both non-targeting treated (abbreviated as control) and in siRNA-treatments as indicated. Data for each siRNA treatment was normalized to the mean value of the control group (black horizontal line). Percentages indicate the drop in the mean value of the siRNA-treated group when compared to the control group for each indicated treatment. Number of cells: 9 in control PRC1, 6 in PRC1 siRNA, 13 in control KIF4A, 13 in KIF4A siRNA, 13 in control MKLP1, 18 in MKLP1 siRNA, 9 in MKLP1+MKLP2 siRNA, 14 in control MKLP2, 11 in MKLP2 siRNA, 6 in control KIF14, 5 in KIF14 siRNA, 23 in control KIF18A, 19 in KIF18A siRNA, 13 in control HSET and 11 in HSET siRNA. At least two independent experiments for every condition regarding siRNAs or non-targeting treatments were done. (**C**) Live images of control and PRC1 siRNA-depleted RPE-1 cells show longer metaphase and anaphase spindles after PRC1 depletion, similar to previous observations (Pamula, et al., 2019). Time zero represents anaphase onset. (**D**) Live images of HeLa cells with stable expression of MKLP1-GFP from bacterial artificial chromosome (BAC) in control and PRC1 depletion by siRNA (left). Live images of HeLa cells with stable expression of KIF4A-GFP from BAC in control and PRC1 depletion by siRNA (right). Cells were stained with 100 nM silicon rhodamine (SiR)-tubulin (not shown). Images are single z-planes. Time zero represents anaphase onset. Note: the chromosomal pool of KIF4A is not perturbed after depletion of PRC1 protein. Scale bar, 5 µm. (**E**) Immunoblot analysis of lysates from RPE-1 cells stably expressing CENPA-GFP and centrin1-GFP treated with control or indicated siRNAs. Antibodies against the respective proteins were used to validate siRNA mediated knockdown, with GAPDH and beta-actin serving as the loading controls. (**F**) Live images of control and PRC1 siRNA-depleted HeLa cells stably expressing EG5-GFP from a BAC, stained with 100 nM SiR-tubulin (not shown). Images are single z-planes. Time zero represents anaphase onset. Note: EG5 localization to the spindle midzone is not dependent upon PRC1. (**G**) Immunofluorescent images of fixed control and EG5 inhibited (inh.) RPE-1 cells (EG5 inhibited cell was treated with 40 µM STLC for 10 min before fixation) stably expressing CENP-A-GFP and centrin1-GFP (magenta) stained with AlexaFluor594 conjugated with PRC1 antibody (green). Note: PRC1 localization in the spindle midzone is not dependent upon EG5 activity. Scale bars, 2 µm (F-G). (**H**) Live images of PRC1 siRNA depleted EG5 inhibited RPE-1 cell showing perturbed spindle elongation after addition of 40 µM STLC in anaphase. Time zero represents STLC addition. Images are the maximum projection of the acquired z-stack. (**I**) Quantification of kinetochore-to-pole velocities (poleward velocity, n=182 kinetochores in control and 50 in PRC1-depleted STLC treated cells). Statistics: t-test (****P < 0.0001). (**J**) Immunofluorescent images of fixed RPE-1 non-induced PRC1 CRISPR knock-out (KO) and PRC1 CRISPR induced KO cells stained with AlexaFluor594 conjugated with PRC1 antibody (green) and 100 nM SiR-DNA (magenta). Images are maximum projection of acquired z-stack. (**K**) Immunoblot analysis of lysates from inducible CRISPR PRC1 KO RPE-1 cells in control non-induced and after 5-day induction with doxycycline. Antibody against PRC1 was used to validate the extent of the KO, with beta-actin serving as the loading control. (**L**) Plots of relative chromosome segregation distance (Δ) defined as the chromosome-chromosome distance at time t minus the chromosome-chromosome distance at t = 0, over time. Individual chromosome pairs (thin lines), mean (thick grey lines) and SD (grey region) for the indicated conditions. Grey shaded regions represent time frames (0-60s from anaphase onset) in which velocity measurements were performed. Numbers: 14 chromosome pairs in PRC1 KO non-induced, 11 in PRC1 KO and 11 in PRC1 KO treated with 40 µM STLC. Time zero represents anaphase onset. (**M**) Live brightfield images of non-induced PRC1 KO and PRC1 CRISPR KO treated with STLC RPE-1 cells imaged 4 days after doxycycline induction.

Note: DNA was not labelled, and the chromosome segregation defects after STLC treatment were similar to those observed when chromosomes were labelled with SiR-DNA (compare with Figure 2), suggesting that the observed segregation defects were not caused by DNA labelling. White arrowheads indicate position of the chromosome mass. (**N**) Live images of PRC1 KO RPE-1 cells treated with STLC imaged 5 days after doxycycline induction where chromosomes were labelled with 100 nM SiR-DNA (left and middle) and unlabeled cell imaged in brightfield (right). Dashed lines designate the cell borders. Note: massive defects in chromosome segregation were observed when induced PRC1 KO is combined with EG5 inhibition by STLC treatment (compare with Figures 2C and 3C). Time is shown as minutes:seconds. Time zero represents anaphase onset (M, N). Depletion is indicated by an arrow pointing down. Horizontal scale bars, 1 μm. Vertical scale bar, 1min.

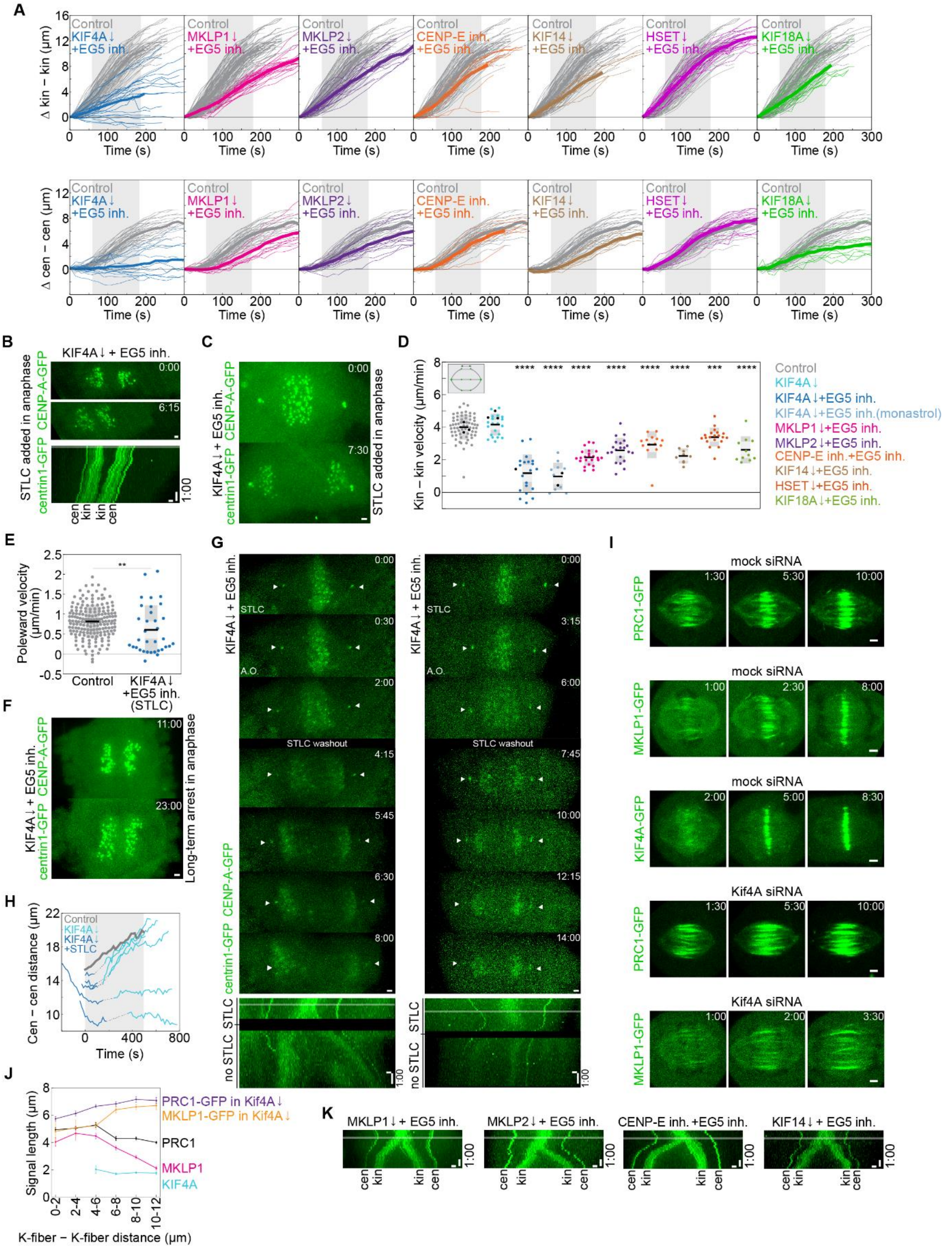


**Figure S3. Depletion of KIF4A combined with EG5 inhibition blocks spindle elongation in anaphase and the effect is reversible if STLC is washed-out during early anaphase, Related to Figure 3.** (**A**) Plots of relative kinetochore and centrosome separation distance (Δ) defined as the kinetochore-to-kinetochore (kin-kin, top row) and centrosome-to-centrosome (cen-cen, bottom row) distance at time t minus the kin-kin and cen-cen distance at t = 0, over time in treatments as indicated. Individual kinetochore and centrosome pairs (thin lines), mean

(thick lines). Time zero represents the onset of anaphase. Grey shaded regions represent the time frame (60-180s from the anaphase onset) in which velocity measurements were performed. Number of kinetochore pairs: 89 in control, 21 in KIF4A-depleted EG5-inhibited, 43 in MKLP1-depleted EG5-inhibited, 8 in MKLP2-depleted EG5-inhibited, 17 in CENP-E and EG5 co-inhibited, 10 in KIF14-depleted EG5-inhibited, 20 in HSET-depleted EG5-inhibited, 10 in KIF18A-depleted EG5-inhibited. Number of cells: 33 in control, 17 in KIF4A-depleted EG5-inhibited, 10 in MKLP1-depleted EG5-inhibited, 12 in MKLP2-depleted EG5-inhibited, 7 in CENP-E and EG5 co-inhibited, 6 in KIF14-depleted EG5-inhibited, 11 in HSET-depleted EG5-inhibited, 10 in KIF18A-depleted EG5-inhibited. At least three independent experiments for every condition regarding siRNAs or non-targeting treatments except KIF14 which was done in two independent experiments, while number of independent experiments regarding STLC and GSK-923295 treatment is equal to the number of cells. (**B**) Live images of KIF4A depleted RPE-1 cell stably expressing CENP-A-GFP and centrin1-GFP (top) and corresponding kymograph (bottom) show perturbed spindle elongation after EG5 inhibition (inh.) by 40 μM STLC addition in anaphase. kin-kinetochore and cen-centrosome. (**C**) Live images of KIF4A siRNA depleted STLC-treated RPE-1 cell, with addition of STLC in anaphase, show small rate of spindle elongation. Note: in a few examples, if STLC was added in early anaphase, spindle elongation could be observed, but to a lesser degree when compared to controls. (D) Quantification of the velocity of separation of sister kinetochores (kin-kin) in the indicated conditions. Statistics: t-test (*P < 0.05, ***P < 0.001; ****P < 0.0001). Note: the effect on kinetochore and centrosome separation velocities seen after depletion or inhibition of PRC1-interacting partners is not modulated further by EG5 inhibition (compare to Figure S1A). Number of kinetochore pairs: 68 in control, 23 in KIF4A depleted , 21 in KIF4A depleted EG5 inhibited, 12 in KIF4A depleted EG5 inhibited with monastrol, 23 in MKLP1 depleted EG5 inhibited, 21 in MKLP2 depleted EG5 inhibited, 14 in CENP-E and EG5 inhibited, 9 in KIF14 depleted EG5 inhibited, 20 in HSET depleted EG5 inhibited, 11 in KIF18A depleted EG5 inhibited. (**E**) Quantification of kinetochore to pole velocity (poleward velocity, n=182 kinetochores in control and 34 in KIF4A-depleted STLC-treated cells). Boxes represent standard deviation (dark grey), 95% confidence interval of the mean (light grey) and mean value (black). (**F**) Live images of KIF4A siRNA depleted STLC-treated RPE-1 cell show long-term anaphase arrest phenotype after STLC addition in anaphase. Note: there is no observable cytokinesis onset for a prolonged time period. Time zero represents the start of STLC treatment. (**G**) Two examples of live images of RPE-1 cells (top) and corresponding kymographs (bottom) of KIF4A siRNA-depleted cells where EG5 was inhibited by treatment with 40 μM STLC in late metaphase and where STLC was washed out (no STLC) by addition of a fresh medium at indicated times (black frames). White arrowheads point to the centriole pair on each side of the spindle. White line in the kymograph indicates the onset of anaphase. A.O., anaphase onset. (**H**) Plots of distance between centrosomes (cen-cen) over time (n=6 cells from three independent experiments). Individual centrosome pairs (full colored lines) after STLC addition (full dark blue lines) and after washout of STLC (full light blue lines) with the mean of the control cells (full thick grey line) during early anaphase (grey region, first 500s after anaphase onset). Last data point before STLC washout and the first data point after STLC washout for each individual cell are connected with a dashed grey line. Time zero represents anaphase onset. Note: the anaphase spindle elongation cannot be reversed by addition of fresh medium if the cell is left in STLC containing medium for more than 3 minutes after anaphase onset. (**I**) Live images of HeLa cells stably expressing PRC1-GFP, MKLP1-GFP and a KIF4A-GFP (green) from a bacterial artificial chromosome (BAC) in a mock siRNA treatment (top 3 rows) and KIF4A siRNA treatment (bottom two rows), stained with 100 nM SiR-tubulin or transiently expressing mRFP-CENP-B (not shown). KIF4A and MKLP1 images: maximum z-projections, PRC1 images: single z-planes. Note: PRC1 labelled bundles, to a greater extent,

and MKLP1 labelled bundles, to a lesser extent, are clearly visible from anaphase onset, whereas KIF4A labelled midzone bundles appear few minutes after anaphase onset. Time zero represents anaphase onset. Scale bars, 2 µm. (**J**) Quantification of the signal length of PRC1-GFP, MKLP1-GFP, and KIF4A-GFP intensities in the spindle midzone with respect to the distance between separating sister k-fibers in a mock siRNA treatment and KIF4A siRNA treatment. 5-pixel-thick line was used to retrieve GFP profile intensities. Every second k-fiber-k-fiber distance corresponds to the columns from E represented by 2-4, 6-8, 10-12 µm distances. Numbers: PRC1: n=6 cells, n(0-2)=13 bundles, n(2-4)=31 bundles, n(4-6)=29 bundles, n(6-8)=22 bundles, n(8-10)=30 bundles, n(10-12)=23 bundles, MKLP1: n=6 cells, n(0-2)=8 bundles, n(2-4)=4 bundles, n(4-6)=16 bundles, n(6-8)=30 bundles, n(8-10)=21 bundles, n(10-12)=27 bundles, KIF4A: n=4 cells, n(4-6)=10 bundles, n(6-8)=17 bundles, n(8-10)=10 bundles, n(10-12)=9 bundles, MKLP1 KIF4A siRNA: n=5 cells, n(0-2)=5 bundles, n(2-4)=5 bundles, n(4-6)=16 bundles, n(6-8)=23 bundles, n(8-10)=24 bundles, n(10-12)=21 bundles and PRC1 KIF4A siRNA: n=7 cells, n(0-2)=32 bundles, n(2-4)=36 bundles, n(4-6)=42 bundles, n(6-8)=35 bundles, n(8-10)=24 bundles, n(10-12)=25 bundles. Number of independent experiments: MKLP1 control: 3, Kif4A control: 1, PRC1 control: 1, MKLP1 Kif4A siRNA: 1, PRC1 Kif4A siRNA: 1. Note: the signal length of PRC1 (Pamula et al., 2019) and MKLP1 labelled antiparallel bundles is decreasing over time, as expected from a system where microtubule sliding is present, although decrease is significantly lower than the net elongation of the spindle (see Figure S1B), implying microtubule polymerization at plus-ends. (**K**) Kymographs made from live images of RPE-1 cells from different perturbations as indicated. Time is shown as minutes:seconds. Horizontal grey lines in the kymographs indicate the onset of anaphase. Images are the maximum projection of the acquired z-stack. Depletion is indicated by an arrow pointing down. Horizontal scale bars, 1 µm. Vertical scale bars, 1min.

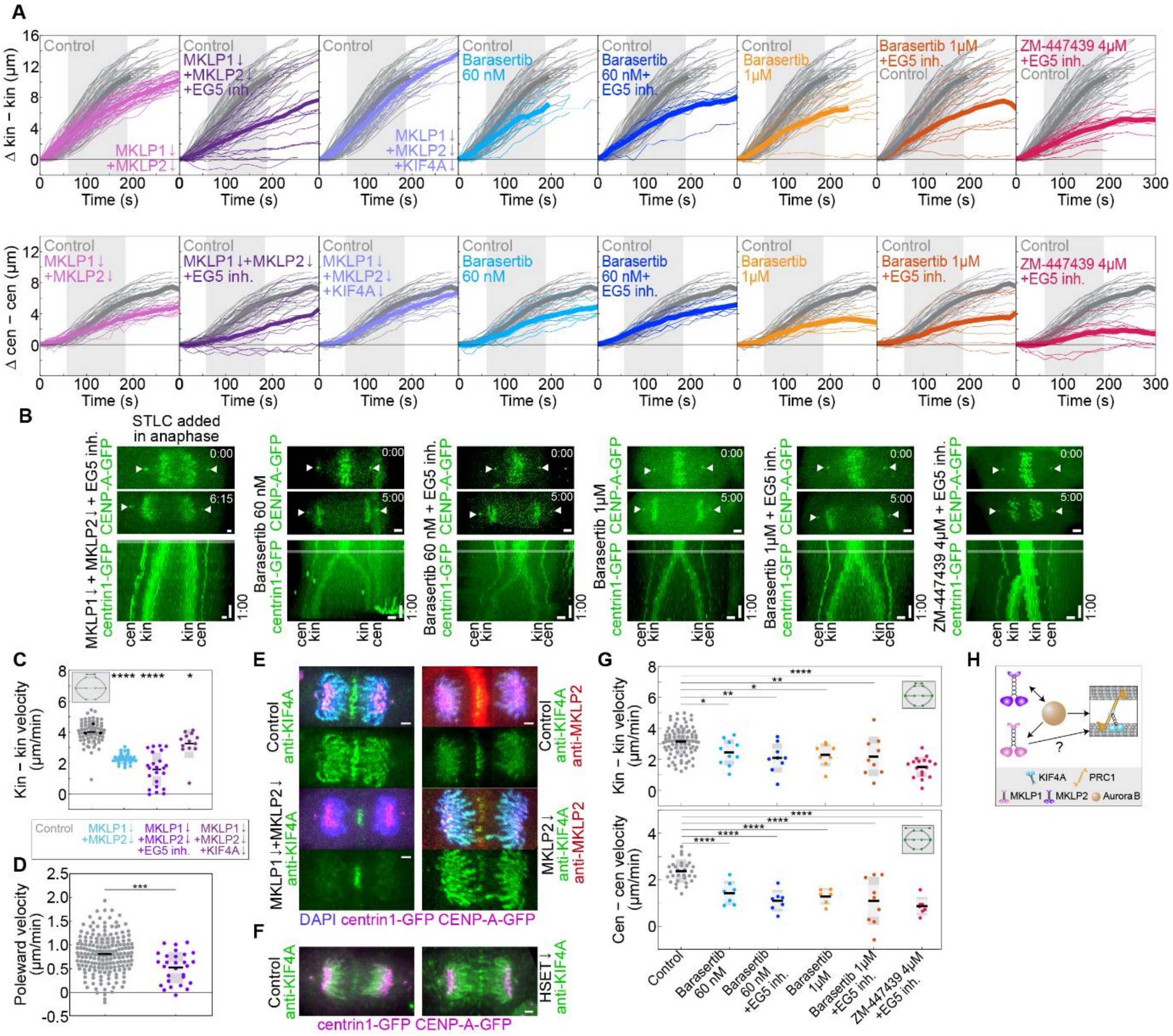


**Figure S4. Depletion of both kinesins-6 when combined with inactivation of EG5 induces partial chromosome segregation block by impairing spindle elongation, but kinesins-6 are not controlling KIF4A midzone localization through Aurora B kinase activity, Related to Figure 4.** (**A**) Plots of relative kinetochore and centrosome separation distance (Δ) defined as the kinetochore-to-kinetochore (kin-kin, top row) and centrosome-to-centrosome (cen-cen, bottom row) distance at time t minus the kin-kin and cen-cen distance at t = 0, over time in indicated treatments. Number of kinetochore pairs: 86 in control, 30 in MKLP1+MKLP2 co-depleted, 21 in MKLP1+MKLP2 co-depleted EG5-inhibited, 15 in MKLP1+MKLP2+KIF4A co-depleted, 10 in 60 nM Barasertib-treated, 8 in 60 nM Barasertib 40 μM STLC co-treated, 9 in 1 μM Barasertib-treated, 10 in 1 μM Barasertib 40 μM STLC co-treated and 17 in 4 μM ZM 40 μM STLC co-treated cells. Number of cells: 32 in control, 12 in MKLP1+MKLP2 co-depleted, 12 in MKLP1+MKLP2 co-depleted EG5-inhibited, 9 in MKLP1+MKLP1+KIF4A co-depleted, 7 in 60 nM Barasertib-treated, 6 in 60 nM Barasertib 40 μM STLC co-treated, 7 in 1 μM Barasertib-treated, 9 in 1 μM Barasertib 40 μM STLC co-treated and 8 in 4 μM ZM 40 μM STLC co-treated cells. Two independent experiments for every condition except MKLP1+MKLP2 co-depleted EG5-inhibited, MKLP1+MKLP2 co-depleted and MKLP1+MKLP1+KIF4A co-depleted cells which were done in three independent experiments, while number of independent experiments regarding drug treatments is equal to number of cells. White line in the kymograph indicates the onset of anaphase. Images are the maximum projection of the acquired z-stack. Individual kinetochore and centrosome pairs (thin lines), mean (thick lines). Grey shaded regions represent the time frame (60-180s

from the anaphase onset) in which velocity measurements were performed. Time zero represents anaphase onset. (**B**) Live images of RPE-1 cells stably expressing CENP-A-GFP and centrin1-GFP (top) and corresponding kymographs from different perturbations as indicated. kin-kinetochore and cen-centrosome. Time zero represents the start of STLC treatment (far left) or anaphase onset (all other panels). Horizontal grey lines in the kymographs indicate the onset of anaphase. Images are maximum projection of acquired z-stack. Time is shown as minutes:seconds. Horizontal scale bars, 1 μm. Vertical scale bars, 1min. (**C**) Quantification of the sister kinetochore separation velocity (kin-kin) in the indicated treatments. Statistics: t-test (*P < 0.05, **P < 0.01; ****P < 0.0001). Number of kinetochore pairs: 67 in control, 31 in MKLP1 + MKLP2 co-depleted, 23 in MKLP1 + MKLP2 co-depleted EG5 treated, 13 in MKLP1 + MKLP2 + KIF4A co-depleted. (**D**) Quantification of kinetochore to pole velocity (poleward velocity, n=182 kinetochores in control and 28 in MKLP1+MKLP2 co-depleted EG5-inhibited cells). Boxes represent standard deviation (dark grey), 95% confidence interval of the mean (light grey) and mean value (black). Statistics: t-test (***P < 0.001). (**E**) Immunofluorescence images of fixed control and RPE-1 cells depleted of target proteins by treatment with indicated siRNAs stably expressing CENP-A-GFP and centrin1-GFP (magenta) stained with AlexaFluor594 conjugated with antibody specific to the indicated target protein (green and red). Chromosomes were stained with 1 μg/mL DAPI solution (blue). (**F**) Immunofluorescence images of fixed control and HSET siRNA-depleted RPE-1 cells stably expressing CENP-A-GFP and centrin1-GFP (magenta) stained with AlexaFluor594 conjugated with antibody specific to KIF4A (green). Scale bars, 2 μm (E, F). (**G**) Quantification of velocity of separation of sister kinetochores (kin-kin, top) and spindle elongation (cen-cen, bottom) in the indicated treatments. Statistics: t-test (*P < 0.05, **P < 0.01; ****P < 0.0001). Number of kinetochore pairs: 86 in control, 10 in 60 nM Barasertib-treated, 9 in 60 nM Barasertib 40 μM STLC co-treated, 8 in 1 μM Barasertib-treated, 9 in 1 μM Barasertib 40 μM STLC co-treated and 17 in 4 μM ZM 40 μM STLC co-treated cells. Note: chromosome segregation and spindle elongation velocity cannot be decreased further by addition of 40 μM STLC when compared to Aurora inhibition alone. (**H**) Proposed model for the regulation of the PRC1-KIF4A motor complex involving both kinesins-6 and Aurora B. Black arrows point to direct or indirect interactions of components in the model. Note: MKLP2 is required for localization of Aurora B (Gruneberg et al., 2004) to the spindle midzone, and Aurora B on the other hand phosphorylates both MKLP1 (Douglas et al., 2010) and MKLP2 (Fung et al., 2017). Aurora B also regulates KIF4A activity by phosphorylation (Nunes Bastos et al., 2013) but MKLP1 is probably also required for the regulation of KIF4A independently of Aurora B-MKLP2 pathway as suggested from observed redundancy between kinesins-6 in this paper.

Depletion is indicated by an arrow pointing down.

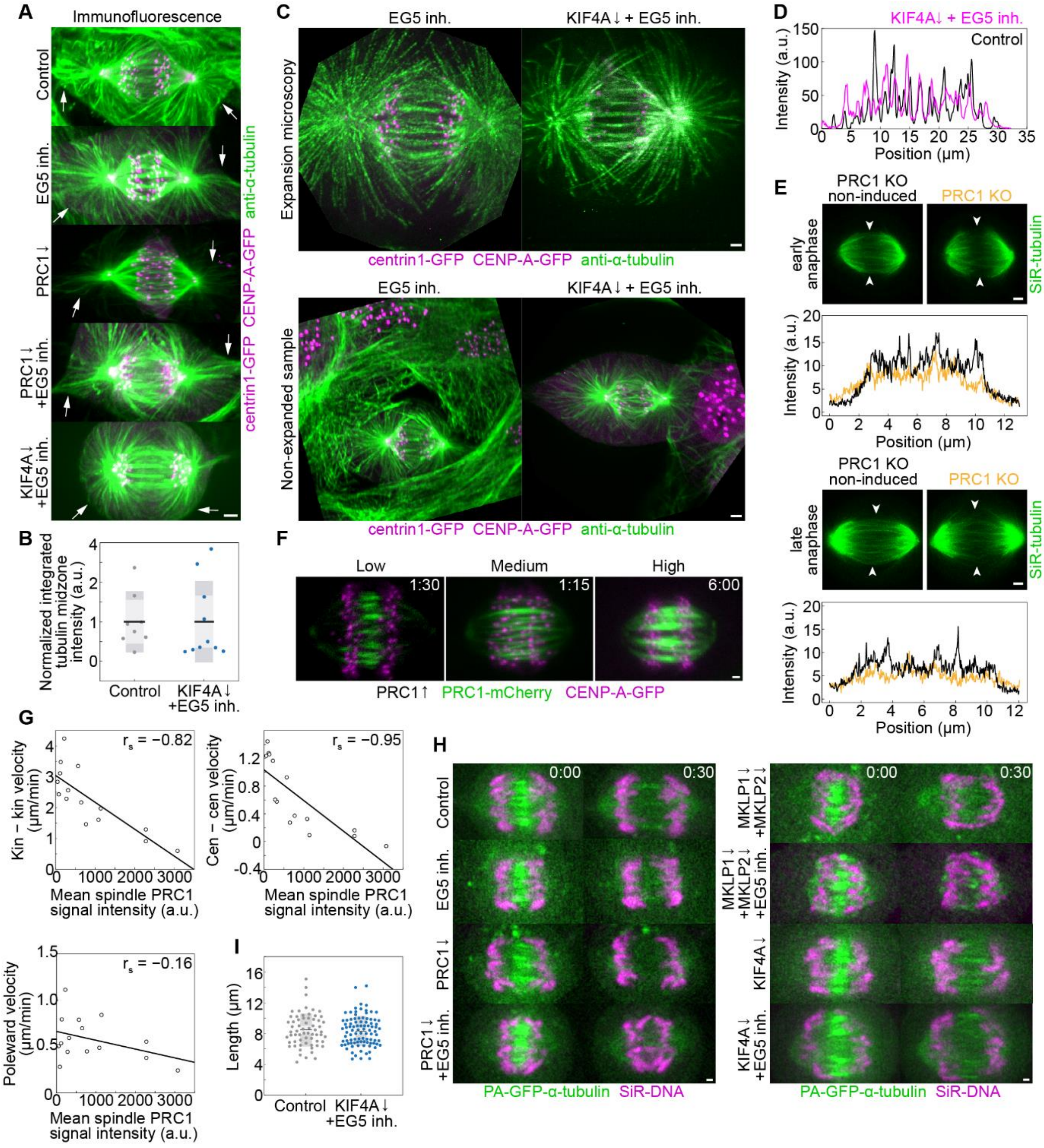


**Figure S5. Perturbation of spindle elongation is not due to the lack of microtubules in the spindle midzone or decreased midzone MT stability during anaphase, whereas spindle elongation can be reduced by overexpression of PRC1 protein, Related to Figure 5.** (**A**) Immunofluorescence images of fixed RPE-1 cells stably expressing CENP-A-GFP and centrin1-GFP (magenta) stained with AlexaFluor594 conjugated with α-tubulin antibody (green) in designated depletions (indicated by an arrow pointing down) and inhibition (inh.) conditions. Arrows point to the place of contact between astral microtubules and the cell membrane. Note: astral MTs reaching the cell borders are observed in all treatments. (**B**) Quantification of normalized integrated tubulin signal intensity in the spindle midzone for the indicated treatments. Boxes represent standard deviation (dark grey), 95% confidence interval of the mean (light grey) and mean value (black). Number of cells: 8 in control and 10 in KIF4A-depleted EG5 inhibited cells. (**C**) Expanded and non-expanded images of fixed 40 µM STLC-treated and KIF4A siRNA-depleted STLC-treated RPE-1 cells stably expressing CENP-A-

GFP and centrin1-GFP (magenta) stained with AlexaFluor594 conjugated with α-tubulin antibody (green). Note: the expansion factor is estimated from the measured spindle length to be 2.3x. Images are maximum projections of the acquired z-stack. (**D**) Signal intensity profiles of α-tubulin from expanded spindles in control and KIF4A-depleted EG5-inhibited cells from Figure 5A measured perpendicular to the spindle midzone. (**E**) STED images (single z-plane) of anaphase spindles in live non-induced control CRISPR PRC1 knock-out (KO) (left) and doxycycline induced PRC1 KO RPE-1 cells (right). Signal intensity profile of SiR-tubulin measured perpendicular to the spindle midzone (bottom graphs). Note: signal intensities after PRC1 KO are slightly decreased and the bundles (signal intensity peaks) are less defined when compared to the non-induced control cells. Scale bars, 2 μm (A-E). (**F**) Live images of U2OS cells stably expressing CENP-A-GFP (magenta) and transiently overexpressing levels of PRC1-mCherry (green) as indicated. Note: high levels of PRC1 protein on the mitotic spindle impair normal elongation of the spindle and normal segregation of chromosomes. Images are maximum projection of acquired z-stack. Time zero represents anaphase onset. (**G**) Linear regression and distribution of kinetochore separation (kin-kin, n=15 cells), centrosome separation (cen-cen, n=15 cells) and kinetochore-to-centrosome (poleward) velocity (n=15 cells) versus mean PRC1 signal intensity. $r_s$, Spearman correlation coefficient, $P < 0.001$ for all parameters. At least three independent experiments for every condition were done. (**H**) Smoothed live images of RPE-1 cells at two time points stably expressing photoactivatable (PA)-GFP-α-tubulin after midzone photoactivation of PA-GFP-α-tubulin (green) in designated protein depletions and protein inhibition conditions. 100 nM SiR-DNA (magenta) was used for chromosome staining. Time zero represents the photoactivation onset. Images are single z-planes. (**I**) Quantification of the length of the astral microtubules from the immunofluorescent images after the indicated treatments. Number of cells: same as in (B). Number of astral microtubules: 64 in control, 92 in KIF4A depleted EG5 inhibited cells. Time is shown as minutes:seconds. Depletion is indicated by an arrow pointing down, while overexpression up. Scale bars, 1 μm (F-H).

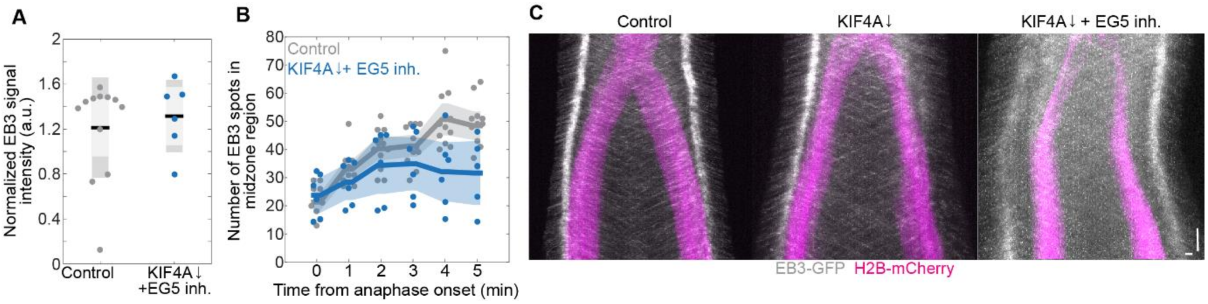


**Figure S6. KIF4A depletion and EG5 inhibition does not impact the number nor the dynamic properties of midzone and astral microtubules, Related to Figure 6.** (**A**) Quantification of integrated intensity of EB3-eGFP signal in the central spindle z-plane in the indicated treatments. Statistics: t-test. Number of cells: 12 in control, 6 in KIF4A depleted EG5 inhibited. (**B**) Graph depicting changes in the number of EB3 comets in the whole midzone in time in both control and KIF4A depleted STLC-treated cells. Note: there are less EB3 comets after KIF4A depletion and EG5 inhibition as these spindles do not elongate their midzones (see Figure 6). Shaded areas represent 95% confidence interval of the mean and thick lines represent the mean values for both indicated treatments. Number of cells: same as in (A). (**C**) Whole spindle kymographs of EB3-GFP (grey) and H2B-mCherry (magenta) expressing RPE-1 cells during 8 min of anaphase in the indicated treatments. Depletion is indicated by an arrow pointing down. Horizontal scale bar, 1 µm. Vertical scale bar, 1min.

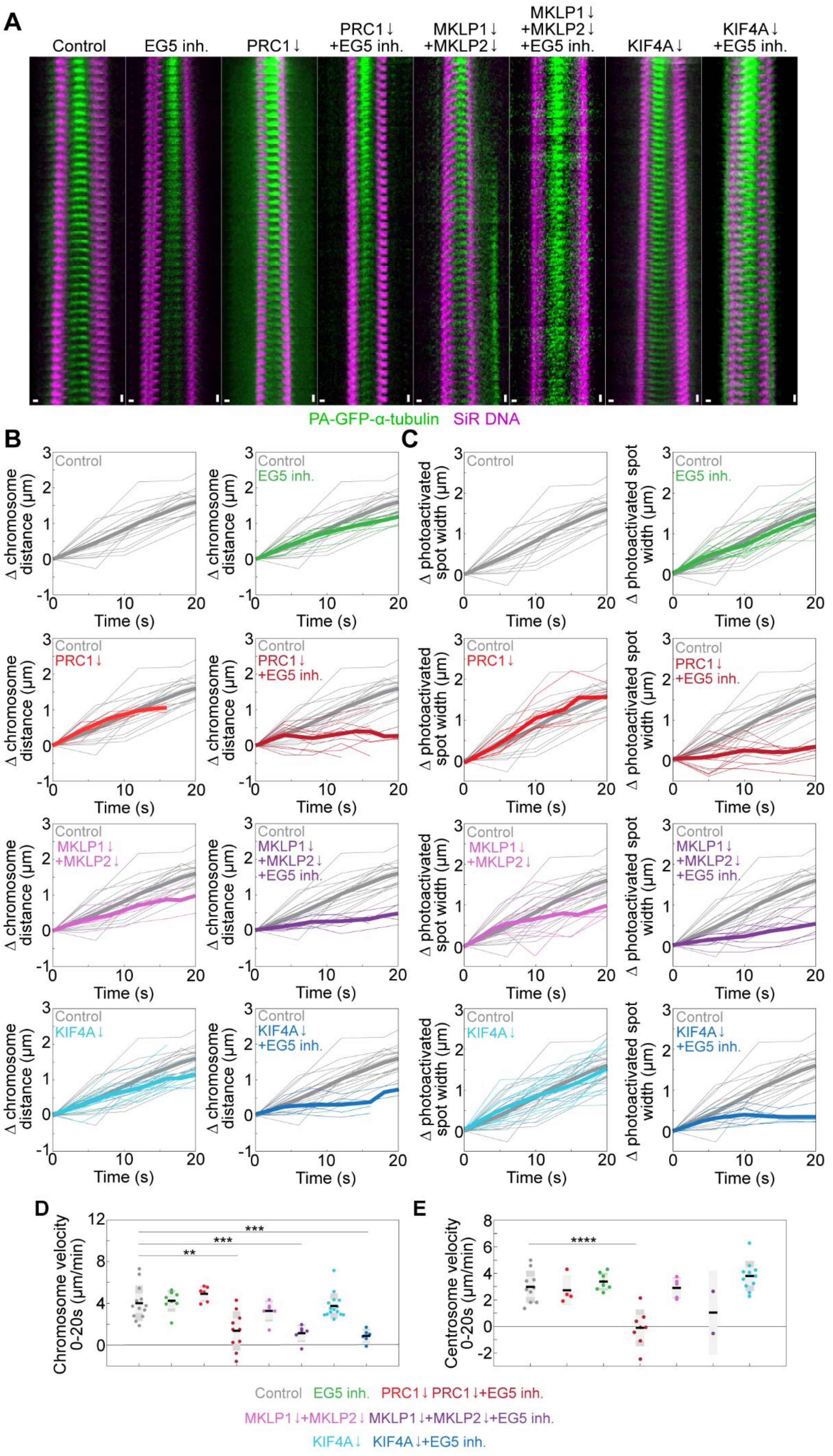
A
Control
EG5 inh.
PRC1↓
PRC1↓ +EG5 inh.
MKLP1↓ +MKLP2↓
MKLP1↓ +MKLP2↓ +EG5 inh.
KIF4A↓
KIF4A↓ +EG5 inh.
PA-GFP-α-tubulin SiR DNA
B
Δ chromosome distance (μm)
Time (s)
C
Δ photoactivated spot width (μm)
D
Chromosome velocity 0-20s (μm/min)
E
Centrosome velocity 0-20s (μm/min)
Control EG5 inh. PRC1↓ PRC1↓+EG5 inh.
MKLP1↓+MKLP2↓ MKLP1↓+MKLP2↓+EG5 inh.
KIF4A↓ KIF4A↓+EG5 inh.

**Figure S7. Sliding of midzone microtubules and anaphase velocities are impaired after EG5 inhibition when combined with conditions perturbing KIF4A motor protein, Related to Figure 7.** (**A**) Smoothed time-lapse live images of single midzone bundles from RPE-1 cells stably expressing photoactivatable (PA)-GFP-α-tubulin after midzone photoactivation of PA-GFP-α-tubulin (green) in in designated protein depletions and protein inhibition (inh.) conditions. 100 nM SiR-DNA (magenta) was used for chromosome staining. Note: the green photoactivated signal is increasing in length during time in most conditions except those where concurrently KIF4A protein was perturbed (PRC1 depletion, MKLP1+MKLP2 co-depletion and KIF4A depletion) and EG5 was inhibited. Vertical scale bars, 0.8s. Horizontal scale bars, 1 μm. (**B**) Plots of relative chromosome segregation distance (Δ) defined as the chromosome-to-chromosome distance at time t minus the chromosome-to-chromosome distance at t = 0, over time in RPE-1 cells stably expressing photoactivatable (PA)-GFP-α-tubulin in the designated protein depletions and protein inhibition conditions. Individual kinetochore and centrosome pairs (thin lines), mean (thick lines). Number of chromosome pairs: 17 in control, 8 in 40 μM STLC-treated, 6 in PRC1-depleted, 11 in PRC1-depleted and STLC treated, 9 in MKLP1+MKLP2 co-depleted, 8 in MKLP1+MKLP2 co-depleted and STLC treated, 15 in KIF4A-depleted and 6 in KIF4A-depleted and STLC treated cells. (**C**) Plots of relative photoactivated spot width (Δ) defined as the photoactivated spot width at time t minus the photoactivated spot width at t = 0, over time in RPE-1 cells in the indicated conditions. Time zero represents the photoactivation onset (B, C). Number of respective bundles measured for sliding velocity: 13 in control, 8 in 40 μM STLC-treated , 6 in PRC1-depleted, 10 in PRC1-depleted and STLC treated, 6 in MKLP1+MKLP2 co-depleted, 6 in MKLP1+MKLP2 co-depleted and STLC treated, 15 in KIF4A-depleted and 6 in KIF4A-depleted STLC treated cells. Three independent experiments for every condition except MKLP1+MKLP2 siRNAs and MKLP1+MKLP2+EG5 inh. which were done in two independent experiments regarding siRNAs or non-targeting treatments, while number of independent experiments regarding STLC treatment is equal to the number of cells. (**D, E**) Quantification of velocity of chromosome segregation (number of cells in each condition corresponds to the B panel) and centrosome separation (number of cells tracked: 10 in control, 4 in PRC1-depleted, 8 in 40 μM STLC-treated, 8 in PRC1-depleted and STLC treated, 5 in MKLP1+MKLP2 co-depleted, 2 in MKLP1+MKLP2 co-depleted and STLC treated and 11 in KIF4A-depleted cells). Boxes represent standard deviation (dark grey), 95% confidence interval of the mean (light grey) and mean value (black) for the indicated conditions. Statistics: t-test (** $P < 0.01$; ***$P < 0.001$; ****$P < 0.0001$). Depletion is indicated by an arrow pointing down.
